# Improved Efficacy of Triple‐Negative Breast Cancer Immunotherapy via Hydrogel‐Based Co‐Delivery of CAR‐T Cells and Mitophagy Agonist

**DOI:** 10.1002/advs.202409835

**Published:** 2025-01-22

**Authors:** Guodong Li, Ruoxin Du, Donghui Wang, Xiangmei Zhang, Lizhuo Wang, Shuangpeng Pu, Xiaoju Li, Shuning Wang, Juliang Zhang, Beichen Liu, Yuan Gao, Huadong Zhao

**Affiliations:** ^1^ State Key Laboratory of Holistic Integrative Management of Gastrointestinal Cancers Biotechnology Center, School of Pharmacy The Fourth Military Medical University Xi'an 710032 P. R. China; ^2^ Department of Thyroid, Breast, and Vascular Surgery Xijing Hospital The Air Force Medical University Xi'an P. R. China; ^3^ Hebei Provincial Cancer Institute Hebei Provincial Key Laboratory of Tumor Microenvironment and Drug Resistance Fourth Hospital of Hebei Medical University Shijiazhuang 050011 P. R. China; ^4^ Center for Mitochondrial Biology and Medicine The Key Laboratory of Biomedical Information Engineering of Ministry of Education School of Life Science and Technology Xi'an Jiaotong University Xi'an 710049 P. R. China; ^5^ Bioinformatics Center of AMMS Beijing 100850 P. R. China; ^6^ Research Institution, Xijing hospital The Fourth Military Medical University Xi'an 710032 P. R. China; ^7^ Department of Hematology, Hebei Provincial Key Laboratory of Tumor Microenvironment and Drug Resistance Fourth Hospital of Hebei Medical University Shijiazhuang 050011 P. R. China; ^8^ Department of General Surgery Tangdu Hospital Air Force Medical University Xi'an 710038 P. R. China

**Keywords:** CAR‐T‐cell therapy, hydrogel co‐delivery, mitophagy agonist, single cell sequencing, TNBC

## Abstract

Leaky and structurally abnormal blood vessels and increased pressure in the tumor interstitium reduce the infiltration of CAR‐T cells in solid tumors, including triple‐negative breast cancer (TNBC). Furthermore, high burden of tumor cells may cause reduction of infiltrating CAR‐T cells and their functional exhaustion. In this study, various effector‐to‐target (E:T) ratio experiments are established to model the treatment using CAR‐T cells in leukemia (high E:T ratio) and solid tumor (low E:T ratio). It is found that the antitumor immune response is decreased in solid tumors with low E:T ratio. Furthermore, single cell sequencing is performed to investigate the functional exhaustion at a low ratio. It is revealed that the inhibition of mitophagy‐mediated mitochondrial dysfunction diminished the antitumor efficacy of CAR‐T‐cell therapy. The mitophagy agonist BC1618 is screened via AI‐deep learning and cytokine detection, in vivo and in vitro studies revealed that BC1618 significantly strengthened the antitumor response of CAR‐T cells via improving mitophagy. Here, injection hydrogels are engineered for the controlled co‐delivery of CAR‐T cells and BC1618 that improves the treatment of TNBC. Local delivery of hydrogels creates an inflammatory and mitophagy‐enhanced microenvironment at the tumor site, which stimulates the CAR‐T cells proliferation, provides antitumor ability persistently, and improves the effect of treatment.

## Graphical abstract

Trop2‐targeted CAR‐T cells experienced reduced efficacy and functional exhaustion at a low E:T ratio caused by mitophagy‐mediated mitochondrial dysfunction. Next, the mitophagy is screened agonist BC1618, which significantly strengthened the antitumor response of CAR‐T cells via improving the level of mitophagy, suggesting a promising approach to increase the efficacy of TNBC immunotherapy.

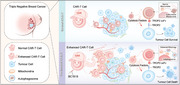

## 1 Introduction

TNBC accounts for 15%−20% of all breast cancer cases and is recognized as the most aggressive subtype with a poor prognosis.^[^ ^1^, ^2^, ^3^ ^]^ Owing to the absence of targetable cell surface proteins such as oestrogen receptor, progesterone receptor, and human epidermal growth factor receptor 2 (HER2),^[^ ^4^, ^5^, ^6^ ^]^ neoadjuvant therapy combined with chemotherapy is currently the main treatment strategy for TNBC patients.^[^ ^7^, ^8^ ^]^ Previous studies have revealed that Trophoblast surface antigen 2 (Trop2) is highly expressed in TNBC as an emerging tumor marker,^[^ ^9^, ^10^ ^]^ and there was a strong correlation between the expression of Trop2 and TNBC progression in this study. Therefore, we developed a chimeric antigen receptor T (CAR‐T) cell therapy that targets Trop2 as a candidate drug for the treatment of TNBC.

To explore and improve the efficacy of CAR‐T‐cell therapy in solid tumor, we established various effector‐to‐target (E:T) ratio experiments to model the effects of CAR‐T‐cell therapy on leukemia (high E:T ratio) and solid tumor (low E:T ratio). It was revealed that effective tumor killing was achieved at a high E:T ratio, but the antitumor immune response decreased at a lower E:T ratio. Leaky and abnormally structured tumor blood vessels and increased pressure in the tumor interstitium both limit the infiltration of immune cells.^[^ ^11^, ^12^ ^]^ Antigen exposure from a substantial tumor burden and persistent CAR signal stimulation can cause functional exhaustion of infiltrating CAR‐T cells.^[^ ^13^ ^]^ Currently, few effective strategies exist to address the functional exhaustion of CAR‐T‐cell therapy in solid tumor.^[^ ^14^ ^]^

To further explore the functional exhaustion of CAR‐T cells in solid tumor, single‐cell sequencing was performed and revealed that the inhibition of mitophagy‐mediated mitochondrial dysfunction diminished the antitumor effects of CAR‐T cells in our study. Mitophagy plays a crucial regulatory role in maintaining mitochondrial homeostasis.^[^ ^15^ ^]^ Mitochondria‐derived metabolic processes, including pyruvate oxidation, the tricarboxylic acid cycle, and one‐carbon metabolism, fulfill the energy requirements of T cells through aerobic respiration, thereby promoting their proliferation.^[^ ^16^ ^]^ Additionally, these processes generate a substantial number of biosynthetic precursors, providing intermediates such as signaling messengers and antioxidants essential for T cell function.^[^ ^16^ ^]^ Damaged mitochondrial related to functional damage, including decrease the generation of ATP, decline the mitochondrial membrane potential, accumulation of mitochondria reactive oxygen species (ROS), inhibited mitochondrial biogenesis, disrupted mitophagy, and subsequently reinforcing T cell exhaustion.^[^ ^17^ ^]^ The large accumulation of ROS promotes T cell exhaustion and impairs their cytotoxic function.^[^ ^18^ ^]^ However, enhanced mitophagy in CAR‐T cells facilitates mitochondrial self‐renewal and ROS scavenging, thereby restoring their antitumor efficacy. Furthermore, BC1618, an inducer of mitophagy in CAR‐T cells, was screened for the first time through AI deep learning and cytokine detection. The level of mitophagy was increased by using BC1618 pretreatment, which reversed the mitophagy inhibition caused by the substantial tumor burden. Finally, we designed a hydrogel‐based release delivery strategy to enhance the sustained antitumor immune response of CAR‐T cells. Collectively, our findings underscore the importance of mitophagy inhibition in CAR‐T‐cell therapy for TNBC, indicating that strategies aimed at amplifying CAR‐T‐cell mitophagy could lead to improved therapeutic outcomes in the treatment of TNBC.

## 2 Experimental Section

### 2.1 Materials

Human breast cancer cell lines including MDA‐MB‐468, HCC1569 were purchased from the Chinese Academy of Sciences (Shanghai, China). X‐VIVO 15 medium was purchased from Lonza (04‐418Q). The rhIL‐2 (200‐02) and rhIL‐15 (200‐15) were purchased from Peprotech. Naïve CD8^+^ T‐cell isolation kit (130‐093‐244) was purchased from Miltenyi Biotec. Red blood cell lysis buffer (00‐4333‐57) was purchased from Thermo Fisher. CryoStorCS10 (100‐1061) solution was purchased from Stemcell. Anti‐human CD3 antibody (317 326) and anti‐human CD28 antibody (302 934) were purchased from Biolegend. TNBC patients tissue samples (BRC1601) were purchased from Shanghai Zhuo Hao Medical Technology Co., Ltd. (Shanghai, China). Rabbit anti‐human Trop2 monoclonal antibody (AB214488) purchased from Abcam. The multiplex IHC kit (PK10006) was purchased from Proteintech. TRIzol (15596026CN) reagent was purchased from Ambio. The HiScriptIIQ RT SuperMix (R323‐01) for qPCR and ChamQ SYBR qPCR Master Mix (Q311‐02) were purchased from Vazyme. RIPA lysis buffer was purchased from Biosharp. Protease inhibitor cocktail was purchased from NCM Biotech. Anti‐scFv antibody was purchased from Genechem. Antimycin A (AMA) and mitophagy agonist library were purchased from MCE. The Elisa kits were purchased from Proteintech. The YoYo1 Green fluorescent dye was purchased from Maokang Biotechnology. Steady‐Glo luciferase substrate (E2510) was purchased from Promega. Mito Tracker Green Dye (201860‐17‐5) and MitoSOX Green Dye (MX4312‐18UG) were purchased from Maokang Biotechnology. Mitophagy detection kit (MD01) was purchased from Dojindo Laboratories. GelMA hydrogels (EFL‐GM‐30), hydrogel lysate (EFL‐GM‐LS), and universal red fluorescent dye (EFL‐GM‐LS) were purchased from Engineering for life.

### 2.2 Cell Culture

The human TNBC cell line MDA‐MB‐468 and the human HER2^+^ breast cancer cell line HCC1569 were cultured in DMEM supplemented with 10% foetal bovine serum (FBS) and 1% 100× penicillin‒streptomycin. Human naïve CD8^+^ T cells and CAR‐T cells were cultured in X‐VIVO 15 complete medium supplemented with 10% FBS, 1% 100X penicillin‒streptomycin, 100 U mL^−1^ rhIL‐2 and 5 ng mL^−1^ rhIL‐15. All the cells were cultured at 37 °C in a humidified 5% CO2 incubator.

### 2.3 Plasmid Construction and Virus Production

Trop2 scFv was generated via phage display technology (KMD Bioscience, Tianjing, China). The CAR plasmid sequence was constructed by fusing the Trop2 scFv to the CD8α hinge and transmembrane domains, 4‐1BB costimulation domain, CD3ζ signaling domain, and SP sequence. Furthermore, to facilitate observation, we constructed an engaged GFP sequence in the Trop2‐targeted CAR plasmid to form a Trop2‐targeted, GFP‐expressing CAR plasmid. Finally, the plasmids were packaged into lentiviruses (Genechem, Shanghai China).

### 2.4 Isolation of Naïve CD8^+^ T Cells

Buffy coats (Air Force Medical University, Shaanxi xi, China) obtained from healthy donors were used to isolate peripheral blood mononuclear cells (PBMCs) via Ficoll‒Paque density gradient centrifugation (GE Healthcare). A 25 mL blood sample was transferred to a 50 mL conical tube and centrifuged at 2500 rpm for 25 min at room temperature. The PBMC layer was aspirated into a fresh 50 mL conical tube and centrifuged at 2000 rpm for 8 min. One milliliter of red blood cell lysis buffer was added to remove the red blood cells. After counting, a naïve CD8^+^ T‐cell isolation kit was used to isolate naïve CD8^+^ T cells according to the manufacturer's instructions. A total of 1 × 10^6^ naïve CD8^+^ T cells were seeded in each well of 24‐well plates and cultured in X‐VIVO 15 complete medium. Naïve CD8^+^ T cells were cryopreserved in CryoStorCS10 solution at concentrations ranging from 1 × 10^7^ to 2 × 10^7^ cells mL^−1^.

### 2.5 Trop2‐Targeted CAR‐T‐Cell Construction

Primary human naïve CD8^+^ T cells were thawed on Day 0 and stimulated with 1 µg mL^−1^ anti‐human CD3 antibody and 1 µg mL^−1^ anti‐human CD28 antibody. After 48 h of CD3/CD28 antibody stimulation, 100 million cells were collected and cultured with X‐VIVO 15 complete medium in each well of 24‐well plates and used to construct CAR‐T cells. Active CD8^+^ T cells were transduced with the lentiviral vectors, 25× HitransG P was added to the total volume of the 1 mL transduction system, and the multiplicity of infection was 20 (MOI = 20). Puromycin (5 µg mL^−1^) selection was used to select CAR‐T cells and CAR‐T‐GFP cells, and control Mock‐T cells were established by transducing active CD8^+^ T cells with empty lentiviral vectors. Quality inspection was performed on Day 10, and the samples were used for the experiments.

### 2.6 Immunohistochemistry (IHC)

We collected 80 paired tumor and peritumoral tissue samples, as well as pathological information from TNBC patients. Four‐micron‐thick paraffin‐embedded TNBC sections were incubated for 2 h in an oven at 60 °C and dried in xylene and gradient ethanol. The sections were immersed in a 98 °C water bath for 18 min in sodium citrate buffer (pH 6.0) for antigen retrieval and quenched with 0.3% hydrogen peroxide. After being immersed, they were washed with phosphate‐buffered saline and blocked with goat serum. The sections were incubated overnight at 4 °C with a rabbit anti‐human Trop2 monoclonal antibody. The sections were washed the next day with PBS and incubated with a secondary antibody for 1 h at 37 °C. A multiplex IHC kit was used for antigen detection according to the manufacturer's instructions. The dehydrated sections were cleared, and haematoxylin was used as a nuclear counterstain. The samples were rehydrated in a gradient series of ethanol solutions, and sealing was performed with gel resin for image analysis. IHC staining for Trop2 was performed by measuring the H score as follows: H score = ∑ Pi × (i + 1), where i represents the intensity score (range 0–4) and Pi represents the percentage of tumor cells stained at each intensity (range 0–100%).

### 2.7 Kaplan‒Meier Plot Analysis

The survival analysis was based on the grading of 80 paired TNBC patient tumor tissue samples. The scoring was stratified into two categories, designated the “high group” and “low group”, based on the median H score.

### 2.8 Reverse Transcription and Quantitative PCR (RT‒qPCR)

Total RNA from the constructed CAR‐T cells was isolated by using TRIzol reagent, followed by reverse transcription into cDNA via the HiScriptIIQ RT SuperMix for qPCR. Then, quantitative PCR assays were performed in triplicate via ChamQ SYBR qPCR Master Mix and processed on a 7500 Fast Real‐Time PCR System (Applied Biosystems, USA). Primers for GAPDH (used as loading controls) and scFv were obtained from Tsingke (Beijing, China) and are listed in **Table** **1**. The relative quantification of RNA expression levels was determined via the 2^(‐ΔΔCT) method.

**Table 1:** The sequence of Primers for GAPDH and scFv.

Primer	Forward (5′‐3′)	Reverse (5′‐3′)
GAPDH	GTCAAGGCTGAGAACGGGAA	AAATGAGCCCAGCCTTCTC
scFv	GTTTCTAGCGGAGGTG GAGG	GATGAGCAGCTTAGG GGCTT

### 2.9 Western Blot Analysis

Cell lysis was performed on ice via RIPA lysis buffer supplemented with a protease inhibitor cocktail. The resulting protein lysates were resolved via SDS‒PAGE and subsequently transferred onto PVDF membranes. The membranes were then blocked with 5% skim milk for 1 h and incubated at 4 °C overnight with specific primary antibodies, including rabbit anti‐human Trop2 monoclonal antibody and anti‐scFv antibody. Following primary antibody incubation, the membranes were washed and incubated with secondary HRP‐conjugated IgG antibodies for 1 h. The protein bands were then visualized and quantified by placing a film on the membrane in a dark room for the appropriate exposure time, followed by incubation with an enhanced chemiluminescence solution.

### 2.10 CAR‐T Cells Cocultured with MDA‐MB‐468 Cells

CAR‐T cells (1 × 10^6^) were seeded in each well of 24‐well plates and cultured for 2 days in X‐VIVO 15 complete medium without rhIL‐2 or rhIL‐15, after which 10 µM AMA, 10 µM drugs from mitophagy library or 10 µM BC1618 was added for pretreatment. Four hundred microliters of a MDA‐MB‐468 cell suspension at a concentration of 1 × 10^6^ mL^−1^ were cocultured with CAR‐T cells at an effector‐to‐target (E:T) ratio of 1: 1 or an E:T ratio of 0.1: 1.

### 2.11 Enzyme‐Linked Immunosorbent Assay (ELISA)

The supernatants of the CAR‐T cells cocultured with MDA‐MB‐468 cells were collected after different treatments. IL‐2, IFN‐γ, and cytotoxic factors, including perforin and GzmB, released into the culture medium were quantified using the respective ELISA kits.

### 2.12 Live Cell Imaging System

CAR‐T cells and mock T cells (5 × 10^4^ cells per 1 mL per well) were cocultured in each well of 24‐well plates with the MDA‐MB‐468 and HCC1569 cells at an E:T ratio of 1:1. YoYo1 Green fluorescent dye (5 µM) (143 413, Maokang Biotechnology) was added to stain dead cells in the coculture medium. Green fluorescence and bright light were captured every 20 min for 24 h via a real‐time cell history recording JuLITM stage imaging system.

### 2.13 Luciferase‐Based Cytolysis Assay

One hundred microliters of MDA‐MB‐468‐luc cells at a density of 1 × 10^6^ mL^−1^ were seeded in a 96‐well opaque white plate and cultured in complete DMEM. CAR‐T cells were subjected to different treatments at the required effector‐to‐target (E:T) ratio and cultured for the indicated times. After the coculture precipitator was collected, the cell precipitates were lysed, and 10 µL of Steady‐Glo luciferase substrate was added to a white opaque plate for 5 min at 37 °C. Luminescence was detected and recorded with a GloMax 20/20 Luminometer. The percentage of specific lysis was calculated via the following equation: % killing = 100 × (RLU from well effector and target cell coculture)/(RLU from well with target cells).^[^ ^19^ ^]^

### 2.14 Single‐Cell RNA Sequencing

CAR‐T cells and MDA‐MB‐468 cells were cocultured at a high E:T ratio (1:1, HR) and at a low E:T (1: 0.1, LR) for three days, and all the cells in the medium were collected on the fourth day. Magnetic‐activated cell sorting (MASC) using CD8 antibody beads was used to separate CAR‐T cells and remove tumor cells. To analyze the droplet‐based data from 10x Genomics, we used the Cell Ranger Single‐Cell software suite (version 7.2.0) provided by 10x Genomics for read alignment and the creation of gene‒cell unique molecular identifier (UMI) matrices for two samples, and we used GRCH38 as a reference genome. The UMI count matrices were then converted into Seurat objects using the Seurat R package (version 4.4.0). Cells meeting quality standards, with detected gene counts ranging from 500 to 5000 and mitochondrial gene ratios under 20%, were retained. Subsequent to the quality assessment, we secured a dataset comprising 31814 cells and 19147 genes for further analysis. Log‐normalized gene expression matrices and linear regression analysis were conducted using Seurat's SCTransform functions. Dimensionality reduction and clustering analysis were performed using principal component analysis (PCA) with Seurat's Run PCA function, while the cell subpopulations were visualized via manifold approximation and projection (UMAP).

### 2.15 Analysis of Gene Set Variations

The analysis of gene set variations was executed via the GSVA package (v1.46.0). Gene sets were sourced from the MSigDB via the msigdbr package. The UCELL package (version 2.2.0) was utilized for calculating functional scores, and the gene sets for estimating the cytotoxicity scores of CD8^+^ T cells were described in the primary research of Shu Zhang et al.^[^ ^20^ ^]^ In order to calculate the cytotoxicity gene signature scores based on scRNA‐seq data, the UCell function was used to score individual cells for cytotoxicity. This function is based on Mann Whitney U statistics to calculate the scores of the selected gene set at the single‐cell level, which are robust to dataset size and heterogeneity. The cytotoxic gene signatures were consisted of genes that translate to effector cytotoxic proteins (GZMA, GZMH, GZMK, GZMM, and NKG7), antigen presentation related genes (HLA‐DPB1, HLA‐DPA1, and HLA‐DRB1), and well‐described cytotoxic T cell activation associated markers in Figure 2C.

### 2.16 Pseudotime Analysis with Monocle2

The developmental trajectories of CD8^+^ T cells were predicted using Monocle2 (version 2.26.0), adhering to the default settings suggested by its developers. The “DDRTree” method was applied for dimensionality reduction in the “reduce Dimension” step. Finally, we employed the “plot_cell_trajectory” function to illustrate the HR and LR groups along a shared pseudotime trajectory.

### 2.17 Electron Microscopy (EM)

For electron microscopy (EM) analysis, CAR‐T cells from the HR and LR coculture groups were fixed in 2.5% glutaraldehyde (EMS) for 1 h at 25 °C. This was followed by postfixation with 1% osmium tetroxide (EMS)/1.5% potassium ferrocyanide (Sigma) for 1 h at 25 °C. After several washes, the samples were dehydrated in acetone (Sigma) and embedded in Epon resin (Sigma). Next, 50‐nm‐thick sections were prepared using a Leica Ultracut microtome (Leica Mikrosysteme), followed by poststaining with 4% uranyl acetate (Sigma) and Reynolds lead citrate (Sigma). Micrographs were captured using a Philips CM100 transmission electron microscope (Thermo Fisher Scientific) at an acceleration voltage of 80 kV with a TemCam‐F416 digital camera (TVIPS). The quantification of normal mitochondria and the enumeration of inner mitochondrial cristae were conducted.

### 2.18 Mitochondrial Function Detection

CAR‐T cells from different coculture groups were collected, centrifuged at 300 × g for 5 min, and the medium was removed with PBS. CAR‐T cells were incubated at 37 °C for 20 min following dilution with 100 nM MitoTracker Green Dye in the absence of serum to label the mitochondria. One micromolar MitoSOX Green Dye was used to incubate CAR‐T cells at 37 °C for 30 min in the absence of serum. The mean fluorescence intensity of all the cells in the samples was subsequently detected via flow cytometry.

### 2.19 Colocalization Analysis on Mitochondria and Lysosomes

A mitophagy detection kit was used to confirm the fusion of mitochondria and lysosomes. CAR‐T cells from different treatment groups were collected, and 100 nM Mtphagy Dye working solution was used to label the mitochondria at 37 °C for 30 min in the absence of serum. The samples were washed with PBS after Mtphagy Dye treatment, and 100 nM Lyso Dye was used to label the lysosomes at 37 °C for 30 min in the absence of serum. The colocalization of mitochondria and lysosomes was detected via laser scanning confocal microscopy.

### 2.20 ATP Assay

Cellular ATP levels were measured by using a firefly luciferase‐based ATP assay kit according to the manufacturer instructions. Briefly, CAR‐T cells were collected in different treatment by centrifuging at 300 g for 5 min. 100 µL of each supernatant was mixed with 100 µL ATP detection working dilution. Luminescence was detected and recorded with a GloMax 20/20 Luminometer.

### 2.21 Mitochondrial Membrane Potential (MMP) Measurement

Changes in MMP were measured using a mitochondrial membrane potential assay kit. Briefly, CAR‐T cells subjected to different treatments were collected and washed twice with cold PBS. The cells were then resuspended in a mixture of 500 µL JC‐1 staining solution and 500 µL medium for 20 min. Subsequently, the CAR‐T cells were washed three times with cold staining buffer and analyzed by flow cytometry.

### 2.22 AI Deep Learning

We employed an artificial intelligence methodology to construct a training dataset comprising 10 000 mitochondrially active compounds. The most efficacious machine learning algorithms were subsequently curated for refinement and training of a classification model. A mitophagy library consisting of 45 mitophagy agonist compounds was obtained from MCE, and the model was then utilized to assess the bioactivity of 45 extant molecules. The top five potential mitophagy agonist were screened according to the score ranking of AI algorithm.

### 2.23 Hydrogel‐Based Drug Delivery Systems

*Hydrogel Formulation and Cell Encapsulation In Vitro*: GelMA hydrogels were prepared in strict accordance with the instructions. 1 × 10^6^ CAR‐T cells were collected and resuspended in the 100 µL hydrogel mixture, and the resulting cell suspension was added to a well plate, gelated by irradiation with a light source at 405 nm for 15 s and cultured in a 5% CO_2_ environment at 37 °C with medium. A 0.3 mg mL^−1^ hydrogel lysate was added to the medium to release CAR‐T cells from the hydrogel.

*Detection of Cell Proliferation and Viability*: The proliferation of CAR‐T‐GFP cells in hydrogel was observed at 0 h and 48 h via laser confocal microscopy. CAR‐T cells proliferation was dynamically monitored by the fluorescence intensity of GFP. Furthermore, CAR‐T cells were cultured in hydrogel on consecutive days and released from hydrogel by hydrogel lysate in various days. All CAR‐T cells were collected to counting and viability detection by placental blue staining.

*Cytokine Release*: CAR‐T cells in the hydrogel were cultured for 5 days and then co‐cultured with tumor cells at a 1:1 effector‐target ratio for 2 days. The cytokine release capacity of CAR‐T cells was measured by Elisa.

*Active Release of CAR‐T Cells*: Low‐concentration hydrogel lysate was added to the medium to simulate the degradation of the hydrogel in vivo. Finally, CAR‐T cells released into medium on different days were collected and counted.

*In Vivo Degradation of GelMA Hydrogels*: To visualize the degradation of the hydrogel in vivo, 5 mg mL^−1^ universal red fluorescent dye (detected at 610 nm) was added to the hydrogel. 100 µL of hydrogel per mouse was injected into BALB/c‐nude mice subcutaneously. Degradation was detected by fluorescence imaging via a fluorescence IVIS system. The experiment was terminated on day 9.

### 2.24 Animal Studies

The experimental animals were 6 weeks female NCG mice (NOD‐Prkdcem26Il2rgem26Gpt) and BALB/c‐nude, obtaining from Gempharmatech (Nanjing, China). All animal experiments were performed in strict accordance with the animal protocol approved by the Air Force Medical University (Shaanxi, China). A 100 µL suspension containing 5 × 10^6^ MDA‐MB‐468 cells was orthotopically inoculated into the mammary fat pad of NCG mice. The orthotopic mice xenografts were divided into four groups for the hydrogel‐based CAR‐T treatment superiority experiment and six groups for the BC1618 pre‐treated CAR‐T antitumor experiment. Each group consisted of five mice with similar tumor volumes. Experiments commenced seven days post‐inoculation, when the tumor size reached ≈100 mm^3^. For the hydrogel‐based CAR‐T treatment superiority experiment, 2 × 10^6^ CAR‐T cells were administered via tail vein injection, intratumor injection, and hydrogel‐based delivery on day 0. For the mitophagy‐enhanced CAR‐T antitumor experiment, 10 µM BC1618 and 2 × 10^6^ CAR‐T cells were mixed with a hydrogel solution. Tumor volumes were measured every five days. After 20 days of treatment, all NCG mice were euthanized, and tumor specimens were harvested for further experimental analysis. BALB/c‐nude mice were used to verify the degradation of hydrogels in vivo.

### 2.25 Statistical Methods

All the data are presented as the means ± SEMs. Statistical analyses were conducted via GraphPad Prism version 8.0 (GraphPad Software). Differences between two groups were assessed via unpaired Student's t tests. For multiple group comparisons, one‐way ANOVA was employed. Survival analysis was performed with the log‐rank test. A *p* value of less than 0.05 was considered indicative of statistical significance.

## 3 Results and Discussion

### 3.1 Engineering Trop2‐Targeted CAR‐T Cells as Potential Therapeutics for TNBC

**Figure 1:**
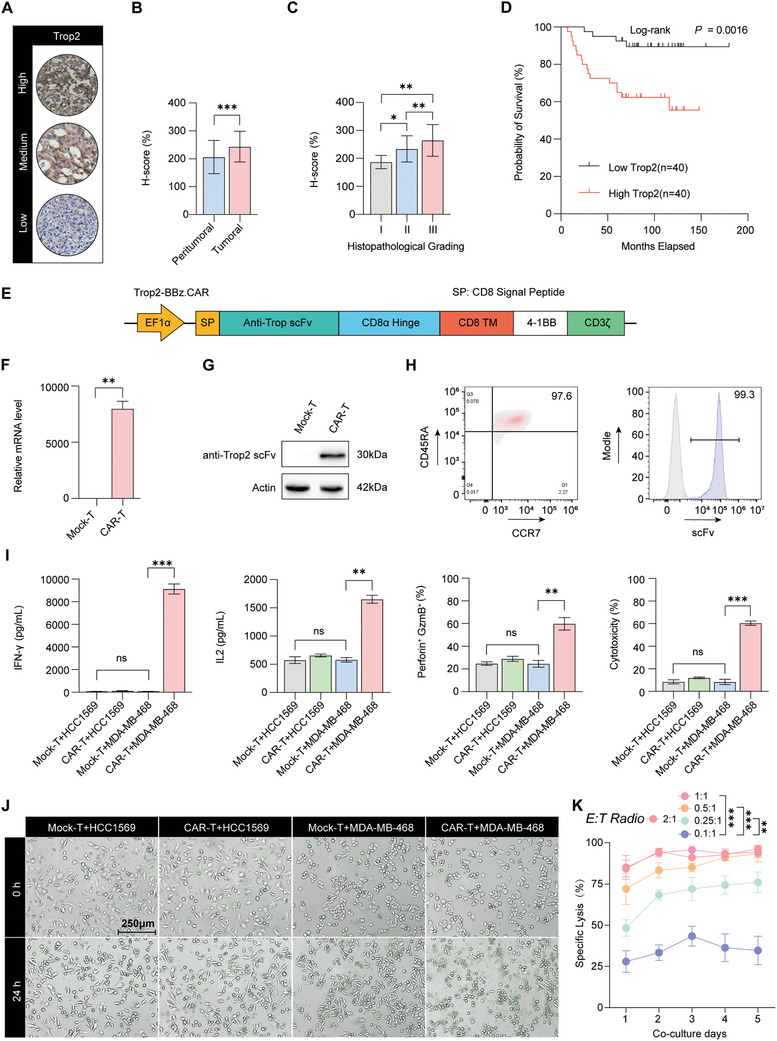
Engineering Trop2‐targeted CAR‐T cells as potential therapeutics for TNBC. A) Representative images of IHC staining for Trop2 expression in TNBC samples, with H‐scores reflecting the intensity and extent of Trop2 expression, and the tissue samples were categorized into high, medium, and low‐expression groups. B–C) Trop2 expression levels in paired tumoral and peritumoral tissues from 80 TNBC patients were assessed by IHC analysis (B), as was the distribution of Trop2 expression across different histological grades and clinical stages of TNBC (C). D) Kaplan‒Meier survival analyses comparing overall survival between 80 TNBC patients with high (n = 40) versus low (n = 40) Trop2 expression. E) Schematic representation of the CAR construct, featuring the SP peptide, Trop2‐targeting scFv, CD8α hinge and transmembrane domains, 4‐1BB costimulation domain and CD3ζ signaling domain. F–G) Quantification of anti‐Trop2 scFv mRNA expression (F) and protein levels (G) in CAR‐T cells, with mock‐transduced T cells (Mock‐T cells) serving as controls. H) Flow cytometric analysis illustrating the phenotype of naïve CD8^+^ T cells defined by the expression of CD45RA and CCR7 and the expression of anti‐Trop2 scFv on the surface of CAR‐T cells after puromycin selection. I) Cytokine production and cytotoxic factor release were measured via ELISA, and the expression of IFN‐γ, IL‐2, granzyme B and cytotoxic factors was detected in the supernatants of cocultures containing Mock‐T cells, CAR‐T cells, and HCC1569 or MDA‐MB‐468 breast cancer cells in the coculture supernatants of different groups, including the Mock‐T‐cell and HCC1569 cell coculture group, the CAR‐T‐cell and HCC1569 cell coculture group, the Mock‐T‐cell and MDA‐MB‐468 cell coculture group and the CAR‐T‐cell and MDA‐MB‐468 cell coculture group. J) Representative merged sequential images were obtained using the Real‐time Cell History Recorder at 0 h and 24 h, while a green fluorescent dye YOYO1 was added to stain dead tumor cells (scale bar = 250 µm). K) Assessment of CAR‐T‐cell‐mediated cytotoxicity against MDA‐MB‐468‐luc cells at various E:T ratios, including 2:1, 1:1, 0.5:1, 0.25:1, and 0.1:1. (B–C, F, K) The data are shown as the means ± SDs and were analyzed by an unpaired t test. *p < 0.05, **p < 0.01, and ***p < 0.001. (I, J) Data are shown as the means ± SDs and were analyzed by one‐way ANOVA. ns: *p* > 0.05, **p < 0.01, and ***p < 0.001.

To evaluate the expression level of Trop2 in TNBC, we conducted immunohistochemical staining for Trop2 in tissue miceoarrays of 80 paired tumor and peritumoral tissues collected from TNBC patients. The H‐score was calculated by assessing the expression level and area of Trop2, and representative images of the IHC results are shown in **Figure** **1**A. Furthermore, we quantified the expression of Trop2 in both TNBC tumoral tissues and peritumoral tissues and found that Trop2 was markedly upregulated in tumoral tissues compared with peritumoral tissues (Figure 1B). In addition, the expression of Trop2 across different grades and clinical stages of TNBC, which is strongly associated with both the grade and stage of disease, was determined (Figure 1C). Additionally, survival analyses conducted on a cohort of 80 patients with TNBC revealed that a high Trop2 signature was associated with a significantly poorer prognosis than a low Trop2 signature (Figure 1D). The expression of Trop2 was subsequently detected in the TNBC cell line MDA‐MB‐468 and the HER2^+^ cell line HCC1569 by Western blotting, and the results showed that Trop2 expression was greater in MDA‐MB‐468 cells (Figure S1A, Supporting Information). Furthermore, both cell lines were used for subsequent experiments. The high expression of Trop2 in TNBC patients ensures its reliability as a target for treatment, and Trop2‐targeted chimeric antigen receptor T (CAR‐T) cells have been developed for the treatment of TNBC.

Building upon these insights, we constructed Trop2‐targeted CAR‐T cells, which contain a Trop2‐targeting single‐chain variable fragment (scFv), a 4‐1BB costimulatory domain, and an intracellular domain from CD3ζ (Figure 1E). The aforementioned sequence corroborates the robust targeting capacity of Trop2‐positive tumor cells, alongside their direct and swift self‐activation capability. Western blotting and real‐time quantitative PCR (RT‒qPCR) were used to detect the structural integrity of the CAR sequence (Figure 1F–G). The proliferation and in vivo persistence of CAR‐T cells are pivotal factors in tumor eradication and recurrence, and CAR‐T cells derived from naïve sources not only exhibit exceptional antitumor capabilities but also mitigate cytokine release syndrome.^[^ ^21^ ^]^ Thus, naïve CD8^+^ T cells were selected from healthy donor peripheral blood and demonstrated naïve phenotype of T cells by CD45RA and CCR7 marker (Figure 1H). Naïve CD8^+^ T cells were activated by CD3/28 antibody and used to construct Trop2‐targeting CAR‐T cells. CAR‐T cells were further enriched after puromycin screening (Figure 1H). The levels of IFN‐γ, IL‐2, granzyme B, and cytotoxic factors present in the supernatants of CAR‐T cells cocultured with tumor cells (MDA‐MB‐468 and HCC1569) were quantified by ELISA, which revealed high cytokine levels in the coculture group of CAR‐T cells with MDA‐MB‐468 cells (Figure 1I). Furthermore, to intuitively demonstrate the cytotoxic capability of CAR‐T cells, YOYO1 was added to stain dead cells, and a living cell imaging system was used to visualize the killed tumor cells, with videos recorded for each experimental group in Figure 1J, Supplementary Materials 1–4 (Supporting Information), representative statistical data of dead cells in each group are displayed in Figure S1B (Supporting Information). The number of dead tumor cells observed in the videos underscores the robust antitumor efficacy of CAR‐T cells. Both leaky tumor blood vessels with irregular architecture and increased pressure in the tumor interstitium limit the infiltration of immune cells.^[^ ^11^ ^]^ Consequently, only a limited cohort of invasive T cells are able to exert their principal antitumor effects.^[^ ^22^ ^]^ To model the treatment of CAR‐T cells in leukemia (high E:T ratio) and solid tumor (low E:T ratio), we established various E:T ratios and prolonged the coculture period to five days to assess the kinetics of tumor lysis (Figure 1K). CAR‐T‐cell‐mediated tumor cell lysis decreased below 50% after 3 days at a lower E:T ratio (0.1:1, LR). Consequently, we hypothesize that a high tumor cell burden may induce CAR‐T‐cell functional exhaustion; Therefore, overcoming the depletion of CAR‐T cells under high tumor burden may be an effective way to treat solid tumors.

### 3.2 Mitochondrial Dysfunction Reduces the Antitumor Effects of CAR‐T Cells

**Figure 2:**
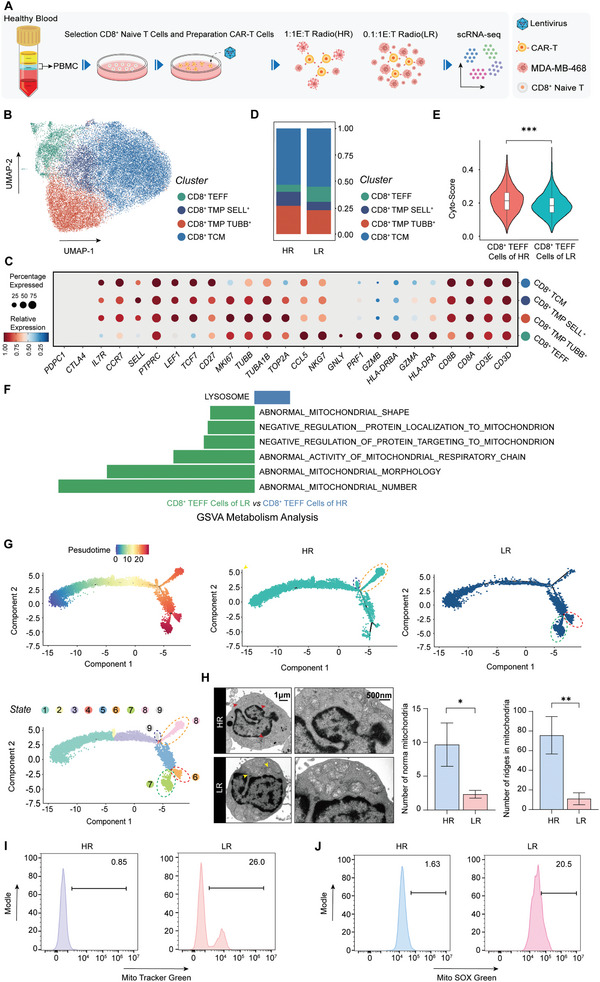
Mitochondrial dysfunction reduces the antitumor effects of CAR‐T cell. A) Preparation for single‐cell sequencing samples HR and LR, including isolation of naïve CD8^+^ T cells from the PBMCs of healthy donors, generation of CAR‐T cells, and coculture with MDA‐MB‐468 cells for 3 days at a 1:1 (E:T) ratio (HR) and 0.1:1 (E:T) ratio (LR). B) UMAP visualizations of single cells from the HR and LR merged samples, colour‐coded according to the four major cell clusters: CD8^+^ TEFF, CD8^+^ TMP SELL^+,^ CD8^+^ TMP TUBB^+^ and CD8^+^ TCM. C) Dot plot displaying the expression of selected genes used for the annotation of the four major cell clusters in HR and LR samples. D). Histogram indicating the relative proportions of the four types of CD8^+^ T cells within the HR and LR samples. E) Violin plot illustrating the Cyto score, a measure of cytotoxic potential, of CD8^+^ TEFF cells in HR and LR samples. F) Gene set variation analysis (GSVA) revealed enrichment of pathways associated with mitochondrial function on the basis of differential gene expression in CD8+ TEFF cells from HR and LR samples. Pseudotime trajectory, with colors ranging from blue to red, representing the developmental progression of T cells in both HR and LR samples. G) The trajectory is divided into nine distinct states, with states 6 and 7 predominantly represented in the LR samples and states 8 and 9 in the HR samples. H) Representative EM images highlighting the structure of mitochondrial reveled by white dotted line, normal f mitochondria are marked by red arrows in the HR sample, abnormal mitochondria are marked by yellow arrows in the LR sample (scale bar = 1 µm), and the structure of inner mitochondrial membranes are showed on the right (scale bar = 500 nm). The numbers of intact mitochondria and inner members of the mitochondria in the HR and LR samples were counted on the right (n = 3). I–J) Flow cytometry revealed the level of ROS in the mitochondria and mitochondrial mass in HR and LR samples via MitoSOX Green and MitoTracker Green, respectively. (H) The data are shown as the means ± SDs and were analysed via an unpaired t test. *p < 0.05, **p < 0.01.

Furthermore, recognizing and addressing the functional exhaustion of CAR‐T cells could enhance the antitumor response. Single‐cell sequencing was subsequently performed on CAR‐T cells cocultured with MDA‐MB‐468 cells at a high ratio (1:1, HR) and a low ratio (0.1:1, LR) for 3 days (**Figure** **2**A). Finally, to ensure the purity of the CAR‐T cells, they were screened by CD8 antibody‐beads isolation after coculture. In conclusion, we constructed CAR‐T cells on the basis of the high expression of Trop2 in TNBC cells and investigated CAR‐T‐cell functional exhaustion in solid tumors via single‐cell sequencing. Initially, CAR‐T cells from the HR and LR groups were identified via uniform manifold approximation and projection (UMAP) visualization (Figure 2B), together with the UMAP plot for detailed information on the different states of the CAR‐T‐cell clusters in Figure 2C. All the cells in the HR and LR merged samples expressed the CD3D, CD3E, CD8A, and CD8B markers. Furthermore, three distinct states of CD8‐positive T cells were defined: effector CD8^+^ T (CD8^+^ TEFF) cells, which highly express perforin (PRF1), granzymes (GZMA, GZMB), and antigen‐presenting genes (HLA‐DRA, HLA‐DRB); central memory CD8^+^ T (TCM) cells, which are characterized by high expression of TCF7, CCR7, and SELL, which is associated with memory cell properties; and memory precursor CD8^+^ T (TMP) cells, which exhibit elevated expression of proliferation‐related genes such as TOP2A and MKI67, alongside memory T‐cell‐associated genes. Then, the memory precursor cells can be subclassified on the basis of differential gene expression into TUBB‐positive and SELL‐positive memory precursor cell subsets. Finally, the contributions of individual cell populations to each analyzed sample are detailed in Figure 2D. Considering that effector CD8^+^ T cells play a main role in the antitumor response, the cytotoxic gene score analysis of CD8^+^ TEFF cells from both the HR and LR samples revealed a lower cytotoxic score in the LR sample than in the HR sample, which aligns with our previous observations concerning the effect‐to‐target ratio experiment (Figure 2E). Intriguingly, the LR sample had a greater number of CD8^+^ TEFF cells, and its cytotoxic activity was reduced (Figure 2D–E). We hypothesize that an increased tumor burden may drive increased differentiation of CD8^+^ T cells into TEFF cells but that persistent CAR signal stimulation may lead to functional exhaustion in CAR‐T cells. To investigate this contradiction, gene set variation analysis (GSVA) was subsequently applied to compare the CD8^+^ TEFF clusters between the two samples (Figure 2F). The results indicated that mitochondrial dysfunction‐related signaling pathways were enriched and significantly different in LR sample and that lysosome‐related signaling pathways were enriched in the HR sample (Figure 2F). Yu et al. revealed the importance of mitochondrial metabolism in the antitumor immune response of CD8^+^ T cells, and mitochondrial dysfunction may directly or indirectly impact the functions of T cells, including their antitumor activity.^[^ ^15^ ^]^ The GSVA results revealed that anomalies in the LR sample included aberrant mitochondrial morphology and quantity, impaired mitochondrial respiratory chain function, and the inhibition of mitochondrial biogenesis (Figure 2F). Therefore, we believed that compared to the HR sample, mitochondrial function was restrained in the LR sample.

Building upon the aforementioned results, we further explored mitochondrial dysfunction in CAR‐T cells in different stages of differentiation in both the HR and LR samples. To trace the developmental trajectories of CAR‐T‐cell lineages, we performed unsupervised cell trajectory analysis via both RNA velocity^[^ ^23^ ^]^ and Monocle2.^[^ ^24^ ^]^ Both analyses revealed similar differentiation paths of CAR‐T cells with two major branches, confirming the accuracy of trajectory prediction (Figure S2A, Supporting Information; Figure 2G). The results suggested that while the cells initially followed similar trajectories, they diverged in later stages, with varying cell abundances between the two samples. Eventually, 9 distinct cell states were identified to represent different stages of differentiation. Notably, the abundance of state 8 and state 9 cells was significantly greater in the HR sample, whereas the abundance of state 6 and state 7 cells was more prevalent in the LR sample. Furthermore, heatmaps based on the state analysis illustrated the differential expression of genes associated with mitochondrial function across these states (Figure S2B, Supporting Information), and states 6 and 7 showed enrichment of signaling pathways involved in mitochondrial dysfunction in the LR sample compared with states 8 and 9 in the HR sample. This enrichment aligns with the GSVA findings, underscoring that mitochondrial dysfunction is prevalent not only in CD8^+^ TEFF cells but also across many cell types in the LR sample. To characterize the state of the mitochondria more directly, mitochondrial morphology from the HR and LR samples was detected via electron microscopy, and morphological examination revealed that the mitochondria in the LR sample exhibited damaged phenotypes, including altered membrane structure, lower crista number, and length of cristae per mitochondrion. The normal mitochondria are marked by red arrows in the HR sample, and the abnormal mitochondrial are marked by yellow arrows (Figure 2H; Figure S2C, Supporting Information). The numbers of normal mitochondria and inner membranes are shown in Figure 2H. Moreover, flow cytometry analysis with MitoTracker Green and MitoSOX Green confirmed an increase in the mitochondrial mass and reactive oxygen species (ROS) levels in the LR sample (Figure 2I–J), further supporting the notion of mitochondrial dysfunction in this group. Compared to the HR sample, there are more tumor burden in LR sample and more persistent antigenic stimulation. Collectively, our findings suggest that persistent antigen stimulation may lead to mitochondrial dysfunction in CAR‐T cells, thereby leading to the reduced antitumor efficacy observed in the context of an increased tumor burden.

### 3.3 Mitophagy Inhibition Mediates Mitochondrial Dysfunction

**Figure 3:**
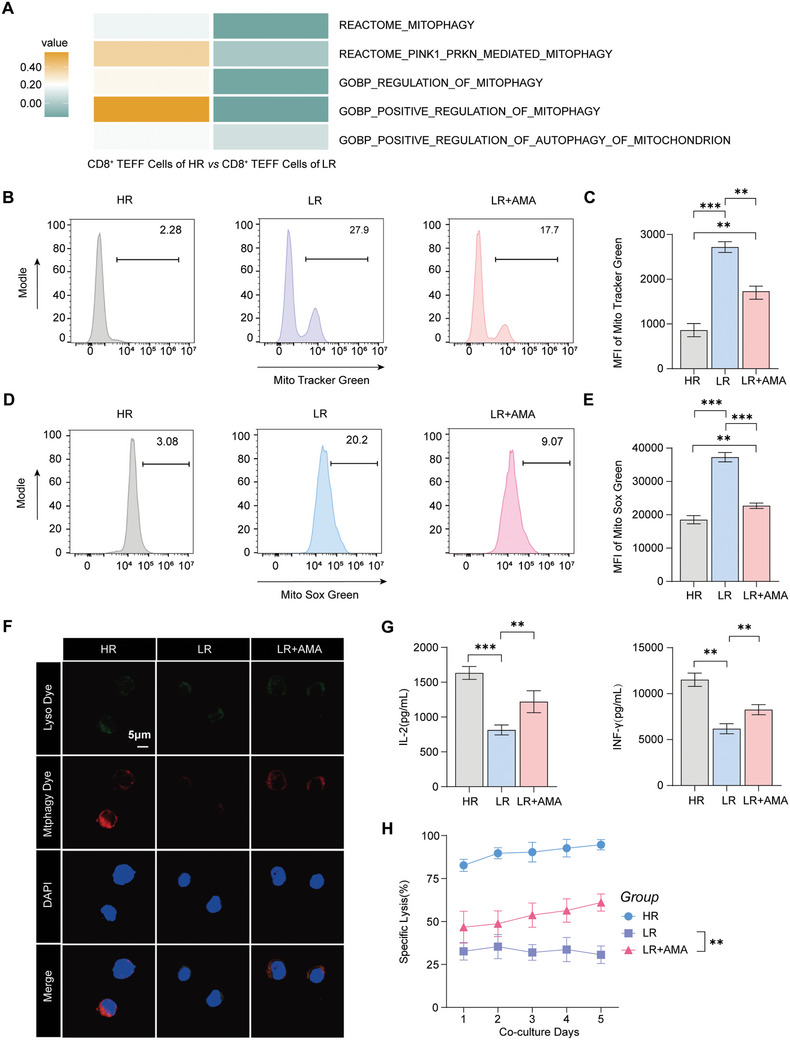
Mitophagy inhibition mediates mitochondrial dysfunction A) Heatmap depicting the enrichment of mitophagy‐associated pathways associated with the DEGs in CD8^+^ TEFF cells from HR and LR samples. B–C) Flow cytometry revealed the mitochondrial mass using MitoTracker Green in the HR, LR and LR AMA pretreatment groups. (B). Furthermore, the mean fluorescence intensity (MFI) of MitoTracker Green was determined in (C), with n = 3. (D–E) Flow cytometry revealed the level of ROS by using MitoSOX Green in the three groups D) Furthermore, the MFI of MitoSOX Green was determined in E), with n = 3. F) Immunofluorescence imaging of mitophagy and mitochondrial‐lysosome colocalization marked by Mtphagy Dye and Lyso Dye, respectively. Nuclei were stained with DAPI; scale bar = 5 µm. G) Cytokine production (IL‐2 and INF‐γ) in the supernatants of cocultured CAR‐T cells and MDA‐MB‐468 cells from the three groups (n = 3). H) Cytotoxic activity of CAR‐T cells against MDA‐MB‐468‐luc cells in the three groups (n = 3). (C, E, G–H) The data are shown as the means ± SDs and were analyzed via an unpaired t test. **p < 0.01 and ***p < 0.001.

Mitophagy plays a crucial role in maintaining mitochondrial quality and function and eliminates damaged mitochondria.^[^ ^25^ ^]^ Previous studies have revealed that impaired mitophagy may result in damaged mitochondria not being removed and may impact T‐cell maturation and differentiation.^[^ ^26^ ^]^ The renewal of mitochondria is also associated with the toxicity of cytotoxic T lymphocytes (CTLs), and inhibiting the biogenesis of mitochondria influences the serial killing function of CTLs.^[^ ^27^ ^]^ Therefore, we employed heatmaps to profile mitophagy‐related pathways in effector CD8^+^ T cells across two samples. Our findings revealed elevated mitophagy in the HR group compared with the LR group, suggesting potential suppression of mitophagy in the LR group (**Figure** **3**A). We further investigated this phenomenon by conducting an enrichment analysis of mitophagy signaling pathways across the entire sample set, with the state analysis used as a reference. The results revealed that mitophagy was reduced in states 6 and 7 from the LR sample compared to states 8 and 9 from the HR sample (Figure S3A, Supporting Information). This observation suggests that a significant tumor burden may provide more persistent antigenic stimulation to CAR‐T cells, leading to inhibited mitophagy and subsequent mitochondrial dysfunction. To investigate the direct relationship between mitophagy and cytotoxicity, a correlation analysis between mitophagy‐associated genes (SREBF2, TSPO, UCP2, and PINK1) and cytotoxicity‐linked genes (NKG7, KLF2, and GZMK) revealed a positive relationship between mitophagy and cellular toxicity (Figure S3B, Supporting Information). These findings further support the hypothesis that mitophagy inhibition may contribute to the diminished antitumor ability of CAR‐T cells.

Drawing on previous research, we postulate that the inhibition of mitophagy caused by continuous antigenic stimulation could be reversed by increasing mitophagy with the use of mitophagy agonists such as antimycin A (AMA). Therefore, CAR‐T cells were pretreated with AMA and subsequently cocultured with MDA‐MB‐468 cells at a lower ratio. The functionality of the mitochondria and the levels of ROS generated by the mitochondrial contribution average fluorescence intensity (MFI) data indicated that the mitophagy agonist successfully enhanced mitophagy in CAR‐T cells (Figure 3B–E). Therefore, the inhibitory effects of antigenic stimulation are effectively reversed by mitophagy agonist pretreatment. To further substantiate the link between mitochondrial function and mitophagy, we employed specific dyes to label lysosomes and mitochondria via Lyso Dye and Mtphagy Dye, respectively. Under physiological conditions, mitochondria and lysosomes maintain their distinct identities (Figure 3F). However, upon the induction of mitophagy, they undergo a series of events culminating in their fusion, which is accompanied by a decrease in pH and results in intense red fluorescence from the mitophagy dye.^[^ ^28^ ^]^ More interestingly, when CAR‐T cells from the LR group were pretreated with AMA, the suppression of mitophagy was alleviated, as evidenced by an increase in the intensity of the red fluorescence (Figure 3F). There was a significant difference in the intensity of red fluorescence (Figure S3C, Supporting Information). Mitophagy is closely correlated with mitochondrial activity; therefore, ATP biosynthesis was assessed in different groups of CAR‐T cells. The results indicated that ATP biosynthesis was increased in AMA‐treated CAR‐T cells compared to the LR group (Figure S3D, Supporting Information). We measured mitochondrial membrane potential from different states of CAR‐T cells, including HR, LR, and the treatment of mitophagy agonist. In damaged mitochondria, the mitochondrial membrane potential decreases and JC‐1 will exist as a monomer showing green fluorescence. The results of flow cytometry analysis showed that continuous antigenic stimulation declined the membrane potential of mitochondrial in LR group, compared to HR (Figure S3E, Supporting Information). Furthermore, mitochondrial membrane potential will be improved by the treatment of mitophagy agonist (Figure S3E,F, Supporting Information). Furthermore, the impact of mitophagy activation on the production of key cytokines was examined. There was a noticeable increase in the levels of IL‐2 and INF‐γ after pretreatment with AMA (Figure 3G), which are indicative of enhanced T‐cell activation and cytotoxic function. Additionally, the results of the luciferase cell killing assay demonstrated the increased antitumor efficacy of mitophagy agonist‐pretreated CAR‐T cells from the LR group (Figure 3H). In summary, these results suggest that persistent antigenic stimulation resulting from a substantial tumor burden can lead to mitophagy‐mediated dysfunction in CAR‐T cells, ultimately compromising their antitumor efficacy. This adverse effect can be mitigated through pretreatment with mitophagy agonists, which can restore mitochondrial function and increase the therapeutic potential of CAR‐T‐cell therapy.

### 3.4 The Mitophagy Agonist BC1618 was Shown to Enhance the Antitumor Effects of CAR‐T Cells

**Figure 4:**
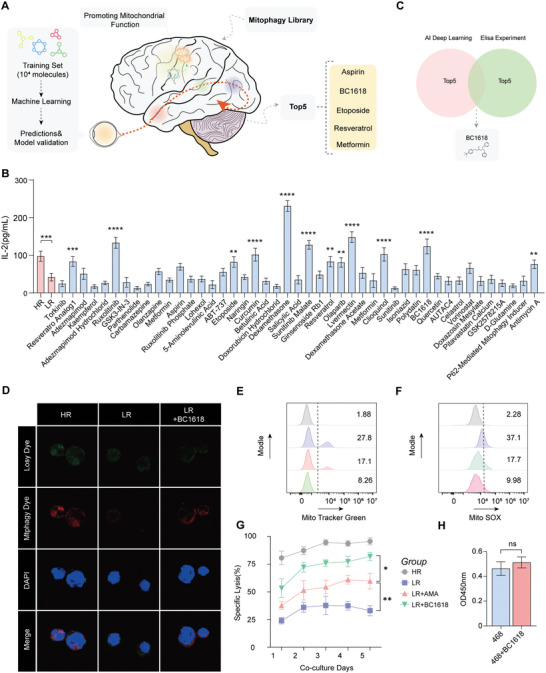
The mitophagy agonist BC1618 was shown to enhance the antitumor effects of CAR‐T cells. A) AI deep learning approach utilized for the systematic screening of potential mitophagy agonists among a dataset of 15 000 mitochondrially active compounds, with a subset of 45 known mitophagy agonists serving as a validation set. B) Experimental validation of the screened candidate agonist through IL‐2 detection by ELISA, (n = 3). C) Selection of BC1618 as the most promising mitophagy agonist on the basis of the AI deep learning and ELISA validation results. D) Immunofluorescence reveals mitophagy activation and mitochondrial and lysosomal colocalization in HR, LR, and LR+A groups pretreated with AMA and LR+BC1618:LR pretreated with BC1618. Representative images for immunofluorescence staining of mitochondria, lysosomes and nuclei stained with DAPI in the HR, LR, LR + AMA, and LR + BC1618 groups. Scale bar = 5 µm. E–F) Flow cytometry revealed the level of mitochondrial mass by using MitoTracker Green (E) and the level of ROS by using the MitoTracker Green (F) in the four groups. G) Cytotoxic activity of CAR‐T cells against MDA‐MB‐468‐luc cells in the four groups (n = 3). H) CCK‐8 assay of the effect of BC1618 treatment on MDA‐MB‐468 cells; n = 3. (B) Data are shown as the means ± SDs and were analyzed by one‐way ANOVA. ns: *p* > 0.05, **p < 0.01, and ***p < 0.001. (G–H) The data are shown as the means ± SDs and were analyzed via an unpaired t test. ns: *p* > 0.05, *p < 0.05, **p < 0.01.

Primary experiments revealed that the cytotoxicity of CAR‐T cells was regulated by mitophagy and that increasing mitophagy promoted the killing effect of CAR‐T cells. However, the application of mitophagy agonists in CAR‐T cells has not yet been reported in CAR‐T cells. To enhance the therapeutic effectiveness of CAR‐T cells against cancer, we established a comprehensive mitophagy library composed of 45 diverse mitophagy agonists. We first used the AI deep learning algorithm to score the mitophagy library, score the top5 mitophagy agonist as shown in **Figure** **4**A and empirical validation via IL‐2 and INF‐γ ELISAs, as shown in Figure 4B and in Figure S4A (Supporting Information), we also pinpointed the five most promising candidates mitophagy agonist. The convergence of these two methodologies led us to select BC1618 as the superior mitophagy agonist, a decision underpinned by its consistent performance in both computational and experimental screens (Figure 4A–C). To achieve a more nuanced understanding of mitophagy dynamics in CAR‐T cells, a dual‐labelling strategy in which lysosomes and mitochondria are selectively labelled with Lyso Dye and Mtphagy Dye was used. Furthermore, the interaction between lysosomes and mitochondria provides a means to quantify mitophagy levels. We observed that following pretreatment of CAR‐T cells with BC1618 and subsequent re‐exposure of tumor cells, there was a notable increase in mitophagy of CAR‐T cells (Figure 4D).

Furthermore, the application of BC1618 to pretreated CAR‐T cells prior to coculture with MDA‐MB‐468 cells resulted in a significant reduction in mitochondrial mass, as evidenced by MitoTracker Green labelling, and a concomitant decrease in ROS levels, as indicated by MitoSOX staining (Figure 4E–F; Figure S4B–C, Supporting Information). Detection of ATP biosynthesis revealed that BC1618 significantly alleviated the ATP synthesis blockade caused by continuous antigen stimulation (Figure S4E, Supporting Information). BC1618 restores mitochondrial ATP synthesis by promoting mitophagy, thereby meeting the energy demands of the anti‐tumor process. Additionally, the result was further corroborated by JC‐1 measurement of mitochondrial membrane potential, which was restored in BC1618‐treated CAR‐T cells (Figure S4F, G, Supporting Information). Therefore, we propose that BC1618, as a potent mitophagy agonist, can restore the mitochondrial activity of CAR‐T cells by enhancing mitophagy. To validate the functional impact of BC1618 on the antitumor activity of CAR‐T cells, a luciferase reporter gene‐based cell cytotoxicity assay was performed, which revealed that CAR‐T cells pretreated with BC1618 presented enhanced antitumor capabilities (Figure 4G). Finally, to ensure that the observed effects on tumor cells were solely attributable to the ability of BC1618 to increase CAR‐T cell activity and not due to any inherent toxicity, we cocultured MDA‐MB‐468 cells with BC1618 for 48 h. The results confirmed that BC1618 did not affect the growth or proliferation of tumor cells, confirming its safety as a therapeutic agent (Figure 4H). In conclusion, BC1618 was screened as a new mitophagy agonist to increase the antitumor response.

### 3.5 Engineering Hydrogel Therapeutic Systems for Cell Delivery

**Figure 5:**
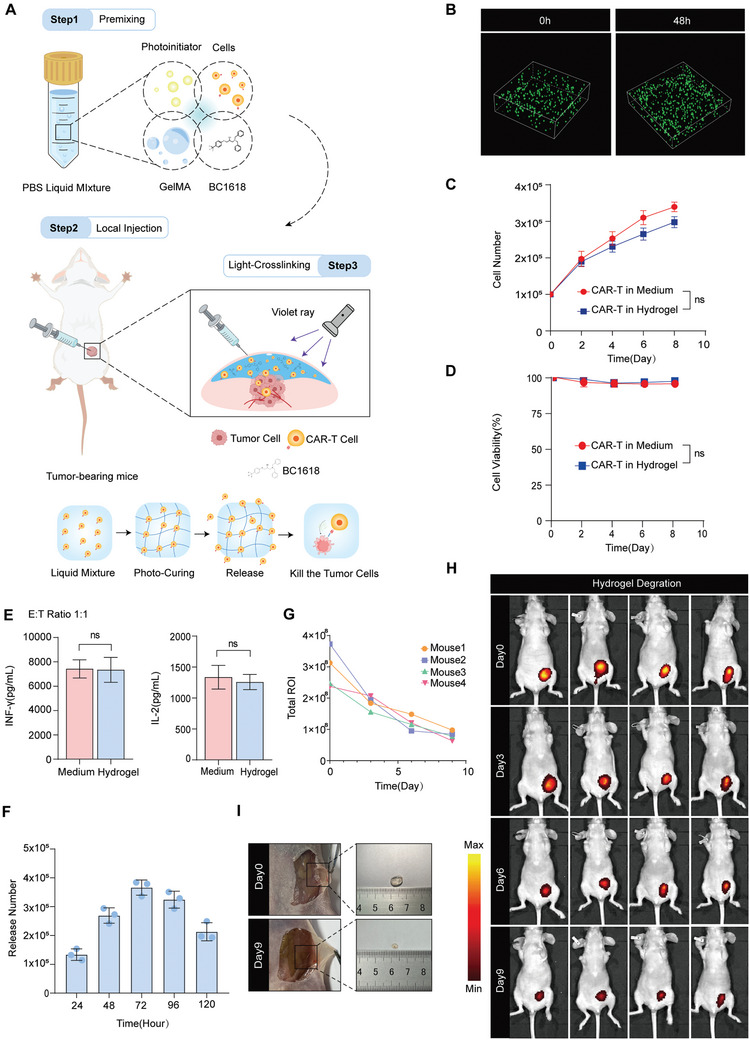
Engineering hydrogel therapeutic systems for cell delivery. A) Schematic diagram of the construction of the injectable hydrogel delivery system. This involves mixing a hydrogel mixture with a photoinitiator, CAR‐T cells, and a mitophagy agonist (BC1618). The delivery system is then injected into the tumor site and subsequent photocuring with violet light at 405 nm is performed, resulting in the formation of a solidified hydrogel matrix embedded with CAR‐T cells that are continuously released into the tumor microenvironment over time. B) Visualization of the growth kinetics of CAR‐T‐GFP cells within the hydrogel over a 48‐hour period, as captured through 3D fluorescence imaging. A total of 1 × 10^5^ CAR‐T‐GFP cells were encapsulated in the hydrogel, and CAR‐T‐GFP cell proliferation was measured via 3D fluorescence imaging at 0 h and 48 h. C) Quantification of the proliferation of CAR‐T cells within the hydrogel compared with that in the culture medium over a 10‐day period. The number of proliferating CAR‐T cells cultured in the medium and hydrogel was counted every 10 days, (n = 3). D) The viability of CAR‐T cells cultured in either medium or hydrogel over a 10‐day period was measured via trypan blue staining, (n = 3). E) The levels of IL‐2 and INF‐γ in CAR‐T cells from medium or hydrogel cocultured with MDA‐MB‐468 cell supernatant (n = 3). F) The number of released CAR‐T cells in vitro was counted, (n = 3). G–H) The fluorescent dye for the hydrogel was added to detect the degradation of the hydrogel in vivo for 9 days (H), and the total ROI was measured (G). Representative images of the subcutaneous hydrogel on Days 0 and 9. (C, D, E) The data are shown as the means ± SDs and were analyzed via an unpaired t test. ns: *p* > 0.05.

Currently, CAR‐T‐cell therapy is predominantly administered via intravenous infusion, which is effective for haematological malignancies but faces challenges in solid tumors because of limited CAR‐T‐cell infiltration and expansion in the immunosuppressive tumor microenvironment.^[^ ^29^ ^]^ In contrast, the deployment of CAR‐T cells through hydrogel vectors markedly increases tumor penetration and antitumor effectiveness.^[^ ^30^ ^]^ The localized delivery strategy minimizes the systemic side effects associated with cytokine release and provides a more conducive environment for CAR‐T‐cell activation and proliferation.^[^ ^31^ ^]^ We developed a hydrogel‐based controlled‐release delivery system for CAR‐T cells. This system involves the dissolution of methacryloyl groups, which serve as precursors, in PBS, along with premixed CAR‐T cells and therapeutic agents, to facilitate the rapid assembly of the delivery platform. When exposed to 405 nm light irradiation for 15 s, the photoinitiator LAP triggers the generation of free radicals, catalyzing the crosslinking of GelMA to form the hydrogel (**Figure** **5**A). To further confirm the practicality of hydrogel‐based delivery, we encapsulated GFP‐expressing CAR‐T cells within the hydrogel and cultured them in medium for 48 h. The results revealed a significant increase in CAR‐T‐cell proliferation within the hydrogel at 48 h compared with that at the initial time point (0 h), indicating the successful encapsulation and sustained viability of the CAR‐T cells (Figure 5B). Furthermore, we encapsulated CAR‐T cells within the hydrogel and systematically dissolved the hydrogel on predetermined days to observe the proliferation patterns of CAR‐T cells in both the culture medium and the hydrogel environment (Figure 5C). CAR‐T‐cell survival was evaluated using trypan blue staining and cell counting (Figure 5D), and the production of cytokines such as IL‐2 and INF‐γ was quantified using ELISA kits (Figure 5E). The collected data confirmed that the hydrogel effectively supported the growth and proliferation of CAR‐T cells, maintaining their biological functions (Figure 5C–E). In summary, hydrogels, as novel biomaterial scaffolds, allow the growth and proliferation of cells while preventing the rapid release of CAR‐T cells, and thus facilitating persistent antitumor effects.

To explore the sustained release properties of the hydrogel, we established both in vivo and in vitro experiments. For the in vitro study, we encapsulated CAR‐T cells within the hydrogel and introduced a low concentration of hydrogel‐dissolving agent to simulate the natural degradation process in vivo. The number of cells released from the hydrogel was continuously monitored over a period of days, revealing a continuous and gradual release profile (Figure 5F). For the in vivo experiments, a specific formulated hydrogel mixed with dyes was injected into a breast fat pad in situ, and the degradation process was monitored over time. The degradation curve and fluorescence In Vivo Imaging System (IVIS) images showed that the hydrogels were gradually hydrolyzed within 9 Days (Figure 5G–H). We further evaluated the characteristics of hydrogels degradation in vivo. As demonstrated in Figure 5I, the hydrogel was separated from the mice after the orthotopic injection of the breast fat pad, hydrogels can be basically degraded at day 9 compared to Day 0. In conclusion, these findings collectively validate the use of hydrogels as efficient delivery systems for CAR‐T cells. This approach not only reduces the quantity of CAR‐T cells required for treatment via allowing the CAR‐T‐cell proliferation, but also enhances the overall safety and efficacy of the therapy by the way of local delivery. The sustained release characteristics of the hydrogel ensure that therapeutic CAR‐T cells are available over an extended period, potentially improving the outcomes of CAR‐T‐cell therapy for patients.

### 3.6 Antitumor Evaluation of Mitophagy‐Enhanced CAR‐T Cells In Vivo

To evaluate whether injection of the hydrogel‐based delivery system can result in greater antitumor efficacy than other administration methods, MDA‐MB‐468 cells were orthotopically implanted into the mammary fat pad of NCG mice and the mice were subjected to various treatments, including CAR‐T cell treatment via tail vein injection or intratumoral injection and the hydrogel‐loaded CAR‐T cell delivery method (Figure S5A, Supporting Information). Tail vein and intratumoral injection of CAR‐T cells elicited a discernible antitumor response, which fully confirmed the antitumor effects (Figure S5B, Supporting Information). However, compared to these injection approaches, that treated with hydrogel‐loaded CAR‐T cells showed significantly reduced tumor size (Figure S5B‐D, Supporting Information). The 3D structure formed by the hydrogel facilitates the proliferation of CAR‐T cells, which are subsequently released into the tumor microenvironment via the degradation of the hydrogel, forming a local inflammatory microenvironment and stimulating the proliferation of the CAR‐T cells, resulting in excellent antitumor effects. Therefore, hydrogel‐loaded CAR‐T cells can be advantageous for realizing sustained antitumor effects.

**Figure 6:**
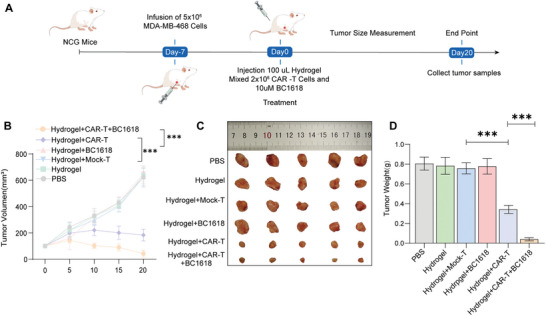
Antitumor efficacy of the hydrogel‐based CAR‐T‐cell release system in vivo. A–D) A murine xenograft model was established by orthotopically injecting 5 × 10^6^ MDA‐MB‐468 cells into the mammary fat pads of NCG mice. Orthotopic mouse xenografts were divided into 6 groups, each containing 5 mice with similar tumor volumes. The mice were then randomly assigned to six experimental groups (n = 5 per group), each with similar initial tumor volumes, as follows: Control: orthotopic injection of 100 µL of PBS. Hydrogel: 100 µL of the hydrogel was orthotopically injected. Hydrogel + Mock‐T: 100 µL of the hydrogel loaded with 2 × 10^6^ Mock‐T. Hydrogel + BC1618: 100 µL of the hydrogel mixed with 10 µM BC1618. Hydrogel + CAR‐T cells: 100 µL of hydrogel loaded with 2 × 10^6^ CAR‐T cells. Hydrogel + CAR‐T cells + BC1618: 100 µL of hydrogel loaded with 2 × 10^6^ CAR‐T cells and mixed with 10 µM BC1618. Treatment was started when the tumor size reached ≈100 mm^3^, and the tumor size was detected every 5 days. (A) The schedule of the animal experiments. (B) The tumor volume in the various treatment groups was determined continuously. (C) Tumor xenografts were extracted and weighed at the end of the corresponding therapies. (D) The quantified data of (C). (B, D) The data are shown as the means ± SDs and were analyzed via an unpaired t test. ns: *p* > 0.05, *p < 0.05, **p < 0.01 and ***p < 0.001.

Furthermore, the results of the primary experiments revealed that the hydrogel‐loaded CAR‐T‐cell strategy has a powerful antitumor effect as a result of BC1618 pretreatment increasing mitophagy, which increased the killing ability of CAR‐T cells in vitro. Consequently, the triple delivery strategy involves a hydrogel, CAR‐T cells and the mitophagy agonist BC1618, which are used for the treatment of TNBC. The experimental protocol is delineated in (**Figure** **6**A). Pretreatment with BC1618 increased mitophagy in CAR‐T cells, and mitophagy enhanced the release of CAR‐T cells into the tumor microenvironment and led to persistent tumor killing. In vivo assays revealed that CAR‐T cells pretreated with BC1618 had increased antitumor activity (Figure 6B–D). In summary, we engineered injection hydrogels for the controlled co‐delivery of CAR‐T cells and mitophagy agonist to enhance CAR‐T‐cell antitumor activity, and a new delivery strategy was used to facilitate the sustained release of CAR‐T cells for a persistent antitumor effect.

## 4 Discussion

Leaky tumor blood cells with irregular architecture and increased pressure in the tumor interstitium limit the infiltration of CAR‐T cells,^[^ ^11^ ^]^ and numerous studies have focused on increasing the infiltration of CAR‐T cells in solid tumors for stronger antitumor effects.^[^ ^32^ ^]^ However, increasing CAR‐T‐cell tumor infiltration may still be insufficient to compensate for the large gap between CAR‐T cells and tumor cells, and CAR‐T‐cell functional exhaustion caused by persistent stimulation of CAR signaling remains unexplored.^[^ ^33^ ^]^ In our study, we revealed that the killing effect was obviously weakened at lower E:T ratios, which reflects CAR‐T‐cell dysfunction in solid tumors. Therefore, overcoming CAR‐T‐cell functional exhaustion and improving the antitumor immune response of single CAR‐T cells constitute a new strategy to address the poor efficacy of CAR‐T‐cell therapy in solid tumors. Through single‐cell sequencing, we revealed that mitophagy inhibition in CAR‐T cells at a lower E:T ratio is a crucial factor leading to CAR‐T‐cell functional exhaustion. Finally, CAR‐T‐cell mitophagy was increased by the use of mitophagy agonists, and the antitumor efficacy of CAR‐T cells was enhanced. To the best of our knowledge, for the first time, we have employed mitophagy agonists to enhance the mitophagy of CAR‐T cells and their antitumor capabilities.

As a type of autophagy, mitophagy allows cells to selectively engulf and degrade damaged mitochondria to maintain mitochondrial homeostasis and the normal biological processes of cells.^[^ ^34^ ^]^ Mitophagy can affect the differentiation and function of T cells by regulating metabolism.^[^ ^17^ ^]^ Mitophagy inhibition leads to insufficient ATP production and impaired cytokine secretion by T cells.^[^ ^35^ ^]^ In our study, inhibition of CAR‐T‐cell mitophagy caused functional exhaustion in CAR‐T cells; furthermore, mitochondrial homeostasis was restored and the antitumor activity of CAR‐T cells was enhanced by increasing mitophagy. Currently, the application of several mitophagy agonists has been explored; for example, metformin induces mitochondrial autophagy via the AMPK pathway for the treatment of diabetic cardiomyopathy.^[^ ^36^ ^]^ However, most mitophagy agonists are still mitochondrial uncouplers or mitochondrial toxins, which have many limitations in terms of clinical efficacy.^[^ ^37^ ^]^ Therefore, screening the mitophagy agonists used in CAR‐T cells is needed. In our study, the optimal mitophagy agonist BC1618 was screened via AI‐based deep learning and cytokine detection analyses and was found to significantly increase mitophagy. BC1618 prevents activated pAmpkα from orphan ubiquitin E3 ligase subunit protein Fbxo48‐mediated degradation to promote mitochondrial fission and facilitate autophagy.^[^ ^38^ ^]^ In summary, BC1618, a mitophagy agonist suitable for CAR‐T cells, was found to enhance the efficacy of CAR‐T cells in this study.

The natural barrier formed by the dense extracellular matrix limits the infiltration of CAR‐T cells and causes poor efficacy of intravenous injection.^[^ ^39^ ^]^ Furthermore, the immunosuppressive microenvironment affects CAR‐T‐cell proliferation and decreases antitumor efficacy after intratumor injection.^[^ ^40^ ^]^ Recent studies have revealed that the local delivery of hydrogels creates an inflammatory microenvironment at the tumor site, allowing the proliferation of CAR‐T cells and enhancing CAR‐T‐cell‐sustained tumor killing.^[^ ^3^ ^]^ In our study, the mitophagy agonist BC1618 combined with a CAR‐T‐cell delivery strategy using a hydrogel was used as a new strategy and demonstrated a more effective therapeutic effect. In vivo experiments revealed that BC1618 increased mitophagy in CAR‐T cells and allowed the slow release of CAR‐T cells to achieve a sustained tumor killing effect.

Gelatin methacryloyl (GelMA), known for its excellent biocompatibility and visible light‐induced crosslinking properties, has been widely used in fields such as 3D cell culture, tissue engineering, and bioprinting, offering promising clinical applications. Breast cancer, being a superficial tumor, is particularly suitable for local drug delivery using hydrogels. This approach ensures precise targeting while avoiding the limitations of systemic circulation, which often leads to low drug penetration and reduced therapeutic efficacy. The potency of BC1618 is approximately 1000 times greater than that of metformin and demonstrates excellent tolerance in mice.^[^ ^38^ ^]^ Furthermore, BC1618 shows outstanding oral bioavailability. Given these properties, the combination of BC1618 delivered via hydrogels and Trop2‐targeted CAR‐T cells offers a promising strategy for the treatment of triple‐negative breast cancer (TNBC) with significant potential for clinical application.

Taken together, we applied BC1618 to promote mitophagy in CAR‐T cells to enhance antitumor effects. A new delivery strategy, BC1618 combined with hydrogel‐based CAR‐T release, provides a new method for TNBC immunotherapies.

## 5 Conclusion

In summary, our research utilized single‐cell sequencing to investigate functional exhaustion of CAR‐T cells, revealing the inhibition of mitophagy‐mediated mitochondrial dysfunction diminished the antitumor efficacy of CAR‐T‐cell therapy. To address the inhibition in CAR‐T cells, we screened the mitophagy agonist BC1618 using AI‐deep learning and cytokine detection. BC1618 significantly enhanced the antitumor response of CAR‐T cells by improving mitophagy levels, suggesting a promising approach to increase the efficacy of TNBC immunotherapy. We engineered injection hydrogels for the controlled co‐delivery of CAR‐T cells and BC1618 that improve treatment efficacy of TNBC.

This approach significantly boosted the antitumor effects of CAR‐T cells, providing valuable insights for the development of effective treatments for TNBC.

## Conflict of Interest

The authors declare no conflict of interest.

## Author Contributions

G.L., R.D., D.W., and X.Z. contributed equally to this work. Y.G., H.Z., and B.L contributed in project conceptualization and supervision. G.L., R.D., D.W., and X.Z. designed and performed the majority of the in vitro and in vivo studies. S.P., X.L., and L.W. provided clinical samples. G.L. and R.D. drafted the manuscript. X.Z. and D.W revised the manuscript. S.W. and J.Z. contributed in funding acquisition. All authors have read and approved the article.

## Supporting information



Supporting Information

## Data Availability

The data that support the findings of this study are available from the corresponding author upon reasonable request.

## References

[1] Y. Li , H. Zhang , Y. Merkher , L. Chen , N. Liu , S. Leonov , Y. Chen , J. Hematol. Oncol. 2022, 15, 121. 36038913 10.1186/s13045-022-01341-0 PMC9422136

[2] S. Al‐Mahmood , J. Sapiezynski , O. B. Garbuzenko , T. Minko , Drug Delivery and Translational Research 2018, 8, 1483. 29978332 10.1007/s13346-018-0551-3 PMC6133085

[3] C. Xu , C. Zhang , K. Ganesan , Q. Chen , H. Tang , F. Gao , Q. Liu , J. Wu , Y. Sui , P. Li , J. Zhang , J. Chen , Curr. Med. Chem. 2023, 31. 10.2174/0109298673259973231023110945 37936460

[4] L. Yin , J. J. Duan , X. W. Bian , S. C. Yu , Breast Cancer Research, BCR 2020, 22, 61. 32517735 10.1186/s13058-020-01296-5 PMC7285581

[5] K. G. K. Deepak , R. Vempati , G. P. Nagaraju , V. R. Dasari , S. Nagini , D. N. Rao , R. R. Malla , Pharmacological Research 2020, 153, 104683. 32050092 10.1016/j.phrs.2020.104683

[6] Y. Tang , W. Tian , S. Zheng , Y. Zou , J. Xie , J. Zhang , X. Li , Y. Sun , J. Lan , N. Li , X. Xie , H. Tang , Research 2023, 6, 0289. 38111678 10.34133/research.0289 PMC10726293

[7] S. Zhu , Y. Wu , B. Song , M. Yi , Y. Yan , Q. Mei , K. Wu , J. Hematol. Oncol. 2023, 16, 100. 37641116 10.1186/s13045-023-01497-3 PMC10464091

[8] X. Ou , Y. Tan , J. Xie , J. Yuan , X. Deng , R. Shao , C. Song , X. Cao , X. Xie , R. He , Y. Li , H. Tang , Drug resistance updates, reviews and commentaries in antimicrobial and anticancer chemotherapy 2024, 73, 101063. 38335844 10.1016/j.drup.2024.101063

[9] M. Cortesi , M. Zanoni , R. Maltoni , S. Ravaioli , M. M. Tumedei , F. Pirini , S. Bravaccini , Expert Opin. Ther. Targets 2022, 26, 593. 35962580 10.1080/14728222.2022.2113513

[10] E. Sakach , R. Sacks , K. Kalinsky , Cancers 2022, 14, 5936. 36497418 10.3390/cancers14235936 PMC9735829

[11] L. Cassetta , J. W. Pollard , Nat. Rev. Drug Discovery 2018, 17, 887. 30361552 10.1038/nrd.2018.169

[12] K. Ganesan , C. Xu , J. Wu , B. Du , Q. Liu , Y. Sui , C. Song , J. Zhang , H. Tang , J. Chen , Science China Life Sciences 2024, 67, 1849. 38900236 10.1007/s11427-023-2499-2

[13] A. H. Long , W. M. Haso , J. F. Shern , K. M. Wanhainen , M. Murgai , M. Ingaramo , J. P. Smith , A. J. Walker , M. E. Kohler , V. R. Venkateshwara , R. N. Kaplan , G. H. Patterson , T. J. Fry , R. J. Orentas , C. L. Mackall , Nat. Med. 2015, 21, 581. 25939063 10.1038/nm.3838 PMC4458184

[14] T. Yan , L. Zhu , J. Chen , Experimental Hematology & Oncology 2023, 12, 14. 36707873 10.1186/s40164-023-00373-7 PMC9883880

[15] Y.‐R. Yu , H. Imrichova , H. Wang , T. Chao , Z. Xiao , M. Gao , M. Rincon‐Restrepo , F. Franco , R. Genolet , W.‐C. Cheng , C. Jandus , G. Coukos , Y.‐F. Jiang , J. W. Locasale , A. Zippelius , P.‐S. Liu , L. Tang , C. Bock , N. Vannini , P.‐C. Ho , Nat. Immunol. 2020, 21, 1540. 33020660 10.1038/s41590-020-0793-3

[16] Y. Huang , X. Si , M. Shao , X. Teng , G. Xiao , H. Huang , J. Hematol. Oncol. 2022, 15, 38. 35346311 10.1186/s13045-022-01255-x PMC8960222

[17] N. J. MacIver , R. D. Michalek , J. C. Rathmell , Annu. Rev. Immunol. 2013, 31, 259. 23298210 10.1146/annurev-immunol-032712-095956 PMC3606674

[18] S. P. Metur , D. J. Klionsky , Cell Mol. Immunol. 2021, 18, 1096. 33785844 10.1038/s41423-021-00662-3 PMC8093269

[19] X. Zhang , C. Zhang , M. Qiao , C. Cheng , N. Tang , S. Lu , W. Sun , B. Xu , Y. Cao , X. Wei , Y. Wang , W. Han , H. Wang , Cancer Cell 2022, 40, 1407. 36240777 10.1016/j.ccell.2022.09.013

[20] S. Lin , Y. Sun , C. Cao , Z. Zhu , Y. Xu , B. Liu , B. Hu , T. Peng , W. Zhi , M. Xu , W. Ding , F. Ren , D. Ma , G. Li , P. Wu , E. Bio. Medicine 2023, 97, 104846. 10.1016/j.ebiom.2023.104846 PMC10618708 37879219

[21] S. Arcangeli , C. Bove , C. Mezzanotte , B. Camisa , L. Falcone , F. Manfredi , E. Bezzecchi , R. El Khoury , R. Norata , F. Sanvito , M. Ponzoni , B. Greco , M. A. Moresco , M. G. Carrabba , F. Ciceri , C. Bonini , A. Bondanza , M. Casucci , J. Clin. Invest. 2022, 132, 150807. 10.1172/JCI150807 PMC9197529 35503659

[22] B. Farhood , M. Najafi , K. Mortezaee , J. Cell. Physiol. 2019, 234, 8509. 30520029 10.1002/jcp.27782

[23] V. Bergen , M. Lange , S. Peidli , F. A. Wolf , F. J. Theis , Nat. Biotechnol. 2020, 38, 1408. 32747759 10.1038/s41587-020-0591-3

[24] L. Haghverdi , M. Büttner , F. A. Wolf , F. Buettner , F. J. Theis , Nat. Methods 2016, 13, 845. 27571553 10.1038/nmeth.3971

[25] G. Ashrafi , T. L. Schwarz , Cell Death Differ. 2013, 20, 31. 22743996 10.1038/cdd.2012.81 PMC3524633

[26] F. Franco , A. Bevilacqua , R.‐M. Wu , K.‐C. Kao , C.‐P. Lin , L. Rousseau , F.‐T. Peng , Y.‐M. Chuang , J.‐J. Peng , J. Park , Y. Xu , A. Cassotta , Y.‐R. Yu , D. E. Speiser , F. Sallusto , P.‐C. Ho , Science Immunology 2023, 8, eadf7579. 37738363 10.1126/sciimmunol.adf7579

[27] M. Lisci , P. R. Barton , L. O. Randzavola , C. Y. Ma , J. M. Marchingo , D. A. Cantrell , V. Paupe , J. Prudent , J. C. Stinchcombe , G. M. Griffiths , Science 2021, 374, eabe9977. 34648346 10.1126/science.abe9977

[28] Y. Huo , W. Chen , X. Zheng , J. Zhao , Q. Zhang , Y. Hou , Y. Cai , X. Lu , X. Jin , J. Cell. Physiol. 2020, 235, 7018. 32083315 10.1002/jcp.29597

[29] A. K. Grosskopf , L. Labanieh , D. D. Klysz , G. A. Roth , P. Xu , O. Adebowale , E. C. Gale , C. K. Jons , J. H. Klich , J. Yan , C. L. Maikawa , S. Correa , B. S. Ou , A. I. d'Aquino , J. R. Cochran , O. Chaudhuri , C. L. Mackall , E. A. Appel , Sci. Adv. 2022, 8, eabn8264. 35394838 10.1126/sciadv.abn8264 PMC8993118

[30] W. Zhou , S. Lei , M. Liu , D. Li , Y. Huang , X. Hu , J. Yang , J. Li , M. Fu , M. Zhang , F. Wang , J. Li , K. Men , W. Wang , Biomaterials 2022, 291, 121872. 36323072 10.1016/j.biomaterials.2022.121872

[31] Q. Hu , H. Li , E. Archibong , Q. Chen , H. Ruan , S. Ahn , E. Dukhovlinova , Y. Kang , D. Wen , G. Dotti , Z. Gu , Nat. Biomed. Eng. 2021, 5, 1038. 33903744 10.1038/s41551-021-00712-1 PMC9102991

[32] M. Martinez , E. K. Moon , Frontiers in Immunology 2019, 10, 128. 30804938 10.3389/fimmu.2019.00128 PMC6370640

[33] A. Chow , K. Perica , C. A. Klebanoff , J. D. Wolchok , Nat. Rev. Clin. Oncol. 2022, 19, 775. 36216928 10.1038/s41571-022-00689-z PMC10984554

[34] Y. Lu , Z. Li , S. Zhang , T. Zhang , Y. Liu , L. Zhang , Theranostics 2023, 13, 736. 36632220 10.7150/thno.79876 PMC9830443

[35] V. M. Hubbard , R. Valdor , B. Patel , R. Singh , A. M. Cuervo , F. Macian , J. Immunol. 2010, 185, 7349. 21059894 10.4049/jimmunol.1000576 PMC3046774

[36] L. Wang , Y. Cai , L. Jian , C. W. Cheung , L. Zhang , Z. Xia , Cardiovasc. Diabetol. 2021, 20, 2. 33397369 10.1186/s12933-020-01188-0 PMC7783984

[37] S. Zhang , W. Fang , S. Zhou , D. Zhu , R. Chen , X. Gao , Z. Li , Y. Fu , Y. Zhang , F. Yang , J. Zhao , H. Wu , P. Wang , Y. Shen , S. Shen , G. Xu , L. Wang , C. Yan , X. Zou , D. Chen , Y. Lv , Nat. Commun. 2023, 14, 5123. 37612267 10.1038/s41467-023-40727-7 PMC10447466

[38] Y. Liu , M. J. Jurczak , T. B. Lear , B. Lin , M. B. Larsen , J. R. Kennerdell , Y. Chen , B. R. Huckestein , M. K. Nguyen , F. Tuncer , Y. Jiang , S. P. Monga , C. P. O'Donnell , T. Finkel , B. B. Chen , R. K. Mallampalli , Nat. Chem. Biol. 2021, 17, 298. 33495648 10.1038/s41589-020-00723-0 PMC8529588

[39] R. C. Sterner , R. M. Sterner , Blood Cancer Journal 2021, 11, 69. 33824268 10.1038/s41408-021-00459-7 PMC8024391

[40] K. M. Maalej , M. Merhi , V. P. Inchakalody , S. Mestiri , M. Alam , C. Maccalli , H. Cherif , S. Uddin , M. Steinhoff , F. M. Marincola , S. Dermime , Molecular Cancer 2023, 22, 20. 36717905 10.1186/s12943-023-01723-z PMC9885707

