# Supplementary material for: Improved Efficacy of Triple‐Negative Breast Cancer Immunotherapy via Hydrogel‐Based Co‐Delivery of CAR‐T Cells and Mitophagy Agonist
Source: Adv Sci (Weinh). 2025 Jan 22;12(14):2409835. doi: 10.1002/advs.202409835 (PMC11984855; doi:10.1002/advs.202409835)
Supplement: Supplementary file 1 — Supporting Information [file ADVS-12-2409835-s001.docx]

**Supplementary materials**


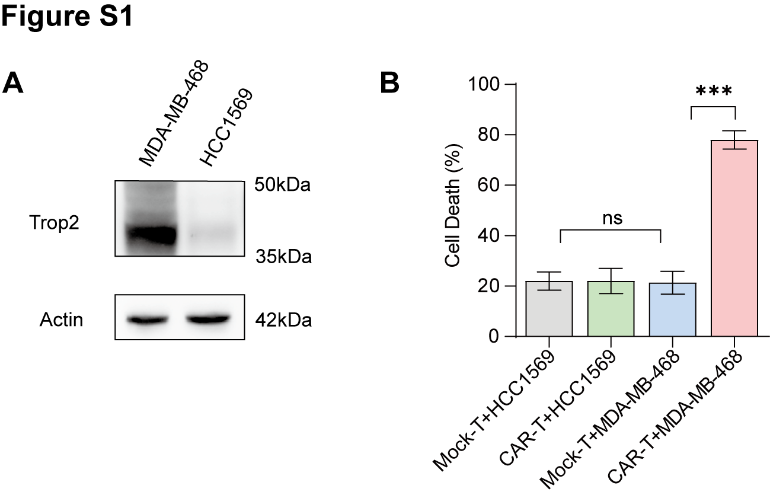


Fig. S1. (A) The protein expression of Trop2 in MDA-MB-468 cells and HCC1569 cells was assessed by Western blotting. (B) Statistical analysis of tumor cell death, with n = 3. The data are shown as the means ± SDs and were analyzed via an unpaired t test, ***p* < 0.01 and ****p* < 0.001.


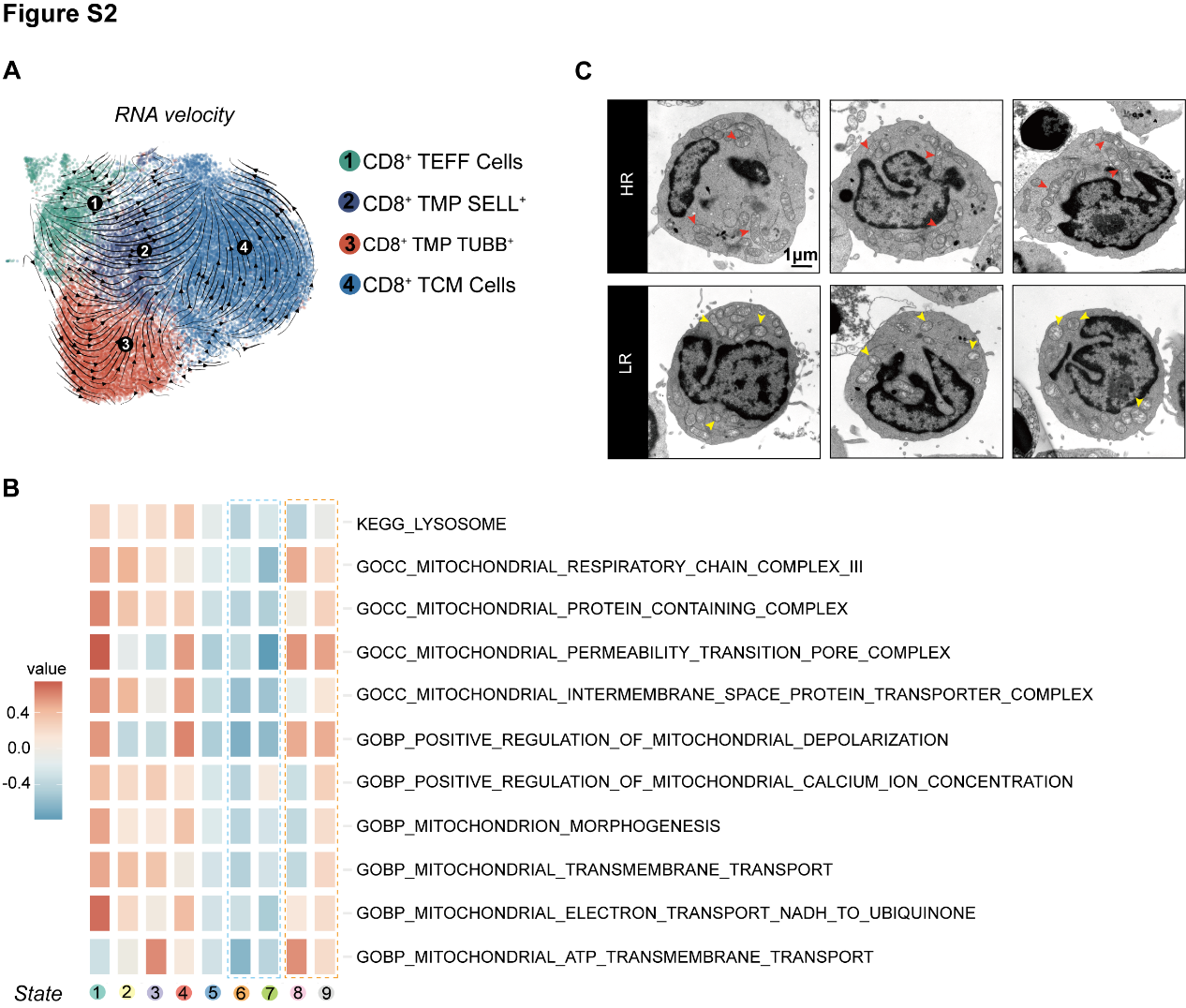


Fig. S2. (A) The RNA velocity revealed the cell lineage from the HR and LR merged samples. Four major cell clusters (CD8^+^ TEFF, CD8^+^ TMP SELL^+^, CD8^+^ TMP TUBB^+^ and CD8^+^ TCM) were identified on the basis of specific marker genes. (B) Heatmap revealing the enrichment of mitochondrial function-associated pathways based on differential gene expression in all HR and LR samples. (C) Representative electron microscopy (EM) images of the HR and LR samples are shown; normal mitochondria in the HR samples are marked by red arrows, and abnormal mitochondria in the LR samples are marked by yellow arrows (scale bar = 1 µm).

**
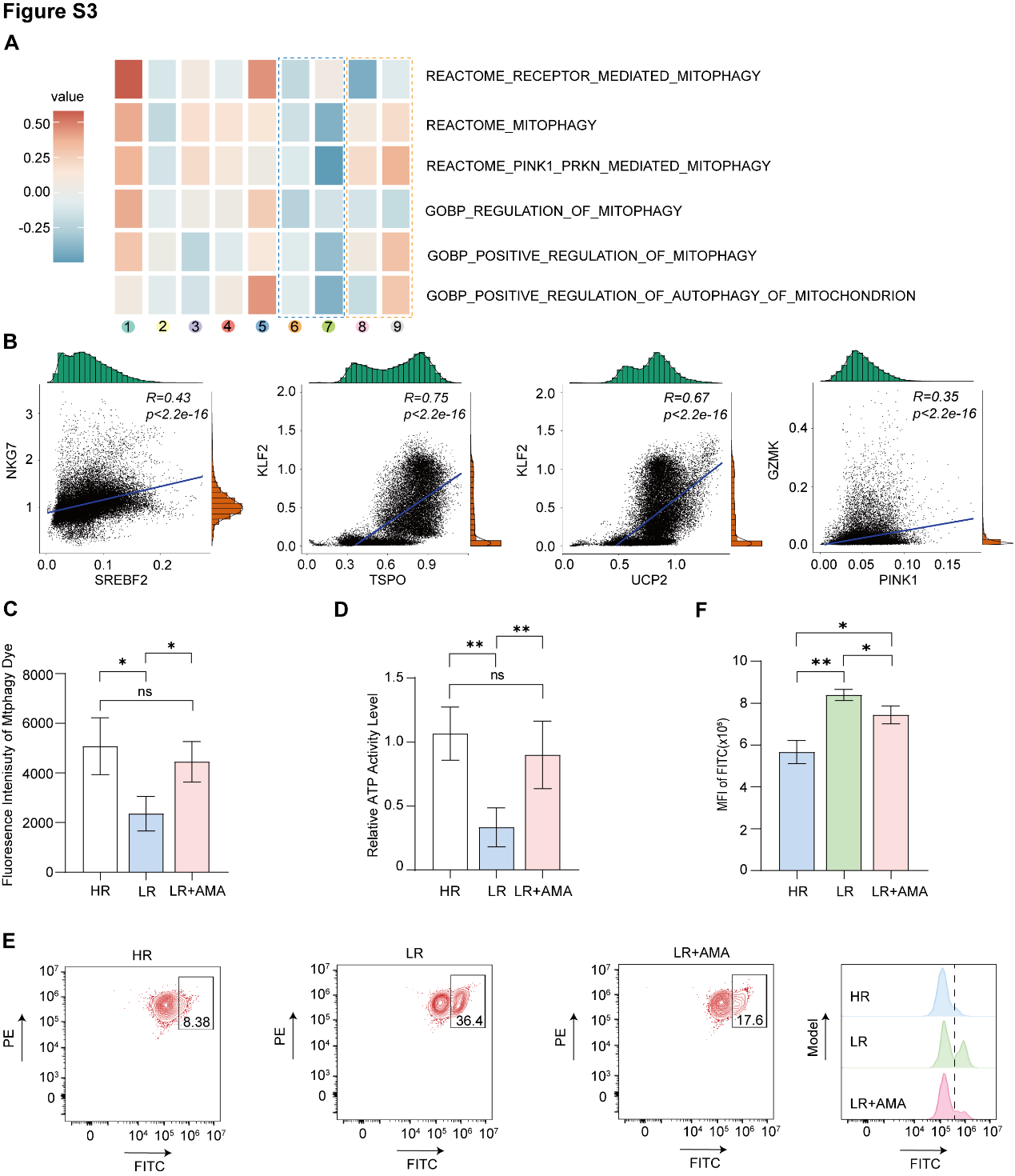
**

Fig. S3. (A) Heatmap showing the enrichment of these pathways across the developmental states of T cells in the merged HR and LR samples, highlighting nine distinct states with varying degrees of mitophagy pathway enrichment. (B) Pearson’s correlation analysis revealing the relationships between the expression of mitophagy-associated genes (SREBF2, TSPO, UCP2, and PINK1) and cytotoxicity-associated genes (NKG7, KLF2, and GZMK) in HR and LR samples. (C) Red fluorescence intensity statistical difference analysis bar graph, with n = 3. (D) Plot of statistical difference analysis of ATP biosynthesis, including HR, LR, LR+AMA groups, with n = 3. (E) Flow cytometry showed the proportion of JC-1 monomers showing green fluorescence. (F) Statistical plot of green fluorescence of JC-1 monomer, with n = 3. (C, D, F) The data are shown as the means ± SDs and were analyzed via an unpaired t test. ***p* < 0.01 and ****p* < 0.001.


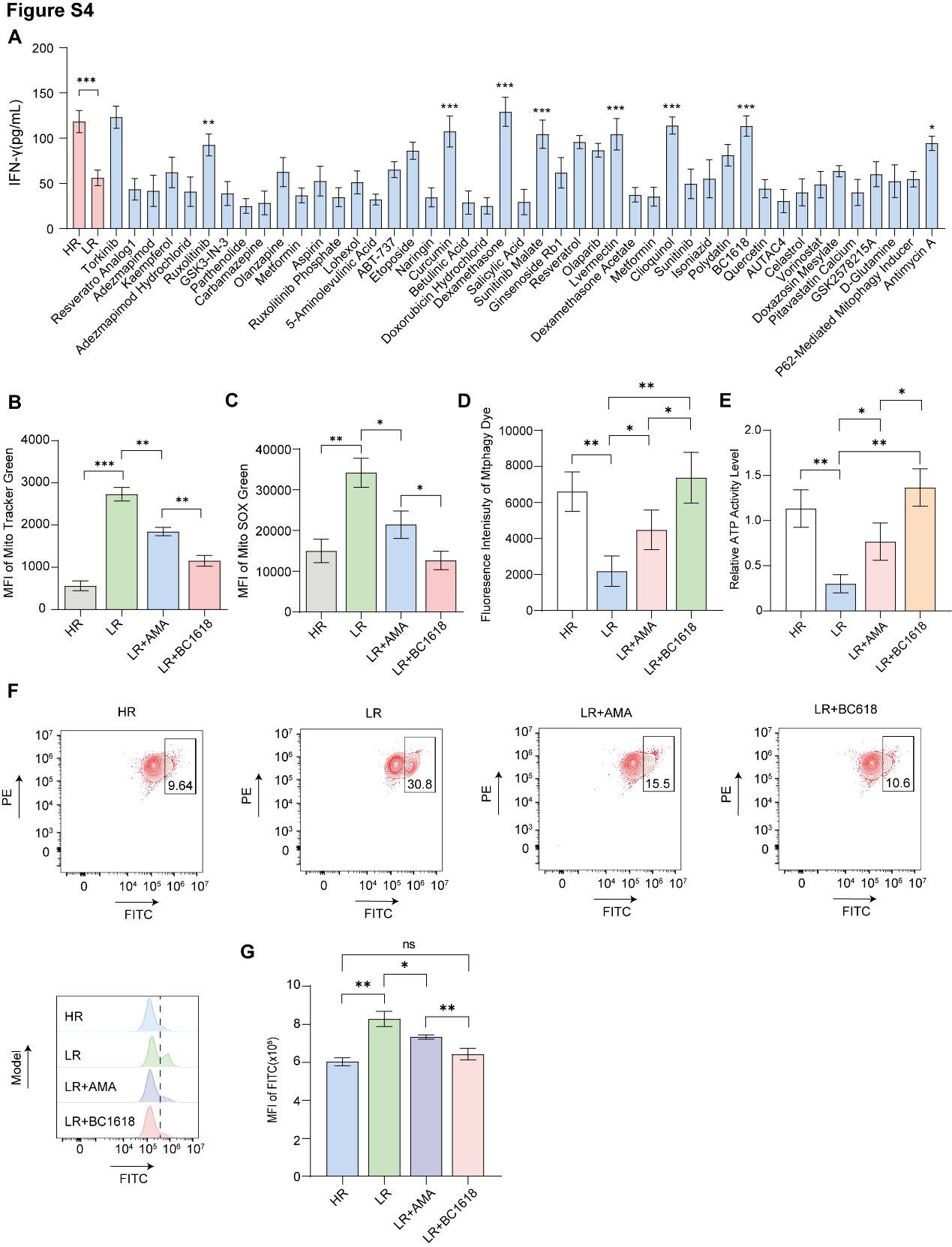


Fig. S4. (A) The mitophagy agonist from the mitophagy library was screened via ELISA to detect the expression of IFN-γ, with n = 3. The data are shown as the means ± SDs and were analyzed via one-way ANOVA. ns: *p* > 0.05, ***p* < 0.01, and ****p* < 0.001. (B-C) Fluorescence statistical analysis of Mito Tracker Green and Mito SOX Green, with n = 3 (D) Red fluorescence intensity statistical difference analysis bar graph, with n = 3. (E) Plot of statistical difference analysis of ATP biosynthesis, including HR, LR, LR+AMA, LR+BC1618groups, with n = 3. (F) Flow cytometry showed the proportion of JC-1 monomers showing green fluorescence. (G) Statistical plot of green fluorescence of JC-1 monomer, with n = 3. (B, C, D, E, G) The data are shown as the means ± SDs and were analyzed via an unpaired t test. ***p* < 0.01 and ****p* < 0.001.


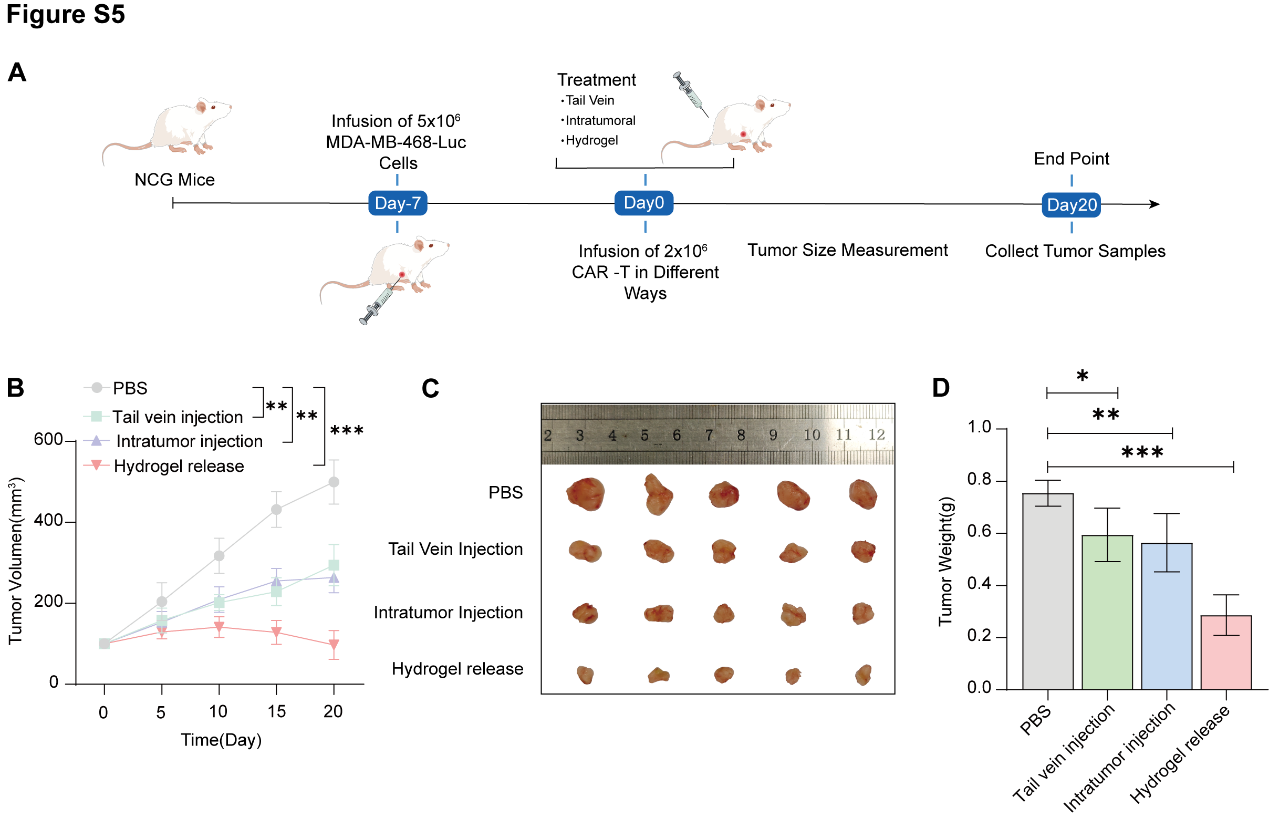


Fig. S5. Compared with tail vein injection and intratumoural injection, hydrogel-based CAR-T-cell therapy revealed optimal antitumour efficiency *in vivo*. (A-D) A total of 5 × 10^6^ MDA-MB-468 cells were orthotopically injected into NCG mice, and orthotopic mouse xenografts were divided into 4 groups, with each group containing 5 mice with similar tumour volumes. Control: 100 μL of PBS was injected via the tail vein. Tain vein injections: 100 μL of PBS containing 2×10^6^ CAR-T cells. Intratumor injection: 100 μL of PBS containing 2×10^6^ CAR-T cells. Hydrogel release: 100 μL of hydrogel loaded with 2×10^6^ CAR-T cells. Treatment was started when the tumour size reached approximately 100 mm^3^, and the tumour size was detected every 5 days. (A) The schedule of the animal experiments. (B) The tumour volume in the various treatment groups was measured continuously. (C) Tumour xenografts were extracted and weighed at the end of the corresponding therapies. (D) The quantified data of (C). (B, D) The data are shown as the means ± SDs and were analyzed via one-way ANOVA. ns: *p* > 0.05, ***p* < 0.01, and ****p* < 0.001.
